# Feeding and food availability modulate brain-derived neurotrophic factor, an orexigen with metabolic roles in zebrafish

**DOI:** 10.1038/s41598-020-67535-z

**Published:** 2020-07-01

**Authors:** Ayelén Melisa Blanco, Juan Ignacio Bertucci, Azadeh Hatef, Suraj Unniappan

**Affiliations:** 1 Laboratory of Integrative Neuroendocrinology, Department of Veterinary Biomedical Sciences, Western College of Veterinary Medicine, University of Saskatchewan, 52 Campus Drive, Saskatoon, SK S7N 5B4 Canada; 2 Laboratorio de Fisioloxía Animal, Departamento de Bioloxía Funcional e Ciencias da Saúde, Facultade de Bioloxía and Centro de Investigación Mariña, Universidade de Vigo, Vigo, Pontevedra Spain; 3 Toxicology Centre, University of Saskatchewan, Saskatoon, SK Canada

**Keywords:** Hypothalamus, Hypothalamus

## Abstract

Emerging findings point to a role for brain-derived neurotrophic factor (BDNF) on feeding in mammals. However, its role on energy balance is unclear. Moreover, whether BDNF regulates energy homeostasis in non-mammals remain unknown. This research aimed to determine whether BDNF is a metabolic peptide in zebrafish. Our results demonstrate that BDNF mRNAs and protein, as well as mRNAs encoding its receptors *trkb2*, *p75ntra* and *p75ntrb*, are detectable in the zebrafish brain, foregut and liver. Intraperitoneal injection of BDNF increased food intake at 1, 2 and 6 h post-administration, and caused an upregulation of brain *npy*, *agrp* and *orexin*, foregut *ghrelin*, and hepatic *leptin* mRNAs, and a reduction in brain *nucb2*. Fasting for 7 days increased *bdnf* and *p75ntrb* mRNAs in the foregut, while decreased *bdnf*, *trkb2*, *p75ntra* and *p75ntrb* mRNAs in the brain and liver. Additionally, the expression of *bdnf* and its receptors increased preprandially, and decreased after a meal in the foregut and liver. Finally, we observed BDNF-induced changes in the expression and/or activity of enzymes involved in glucose and lipid metabolism in the liver. Overall, present results indicate that BDNF is a novel regulator of appetite and metabolism in fish, which is modulated by energy intake and food availability.

## Introduction

Brain-derived neurotrophic factor (BDNF) is a member of the neurotrophin family of proteins isolated from the pig brain in 1982^1^. In fish, cDNAs encoding this peptide have been identified in various species, including the *Danio rerio*^2^, *Anguilla anguilla*^3^, *Cichlasoma dimerus*^4^, and *Nothobranchius furzeri*^5^. The synthesis and maturation of BDNF involve several steps, as well as precursors (pre-pro-BDNF and pro-BDNF) and intermediates, which are sequentially cleaved to form the mature BDNF^6,7^. Both BDNF and pro-BDNF have been shown to exert opposing biological actions in mammals. This suggests that the pro-BDNF/BDNF ratio is an important aspect that determines the regulatory role of this peptide^8^. Synthesis of BDNF in both mammals^9,10^ and fish^4,11–15^ occurs primarily in the brain, specifically in the primary sensory neurons^16^. However, relatively small amounts of this peptide were also detected in the mammalian retina, thymus, heart, lung, gastrointestinal tract, liver, kidney, spleen, reproductive tissues and muscle^17^, and in the fish retina, ear, lateral line, gut and gonads^4,18–23^.

The main receptor mediating BDNF actions is the tropomyosin-related kinase B (TrkB) receptor, which was identified in both mammals^24^ and fish^15^. However, two forms of this receptor, named TrkB1 and TrkB2, have been identified in teleost fish as a consequence of specific genome duplication^25,26^. Between the two isoforms, TrkB2 seems to be the key receptor for BDNF in zebrafish, at least in the brain^27^ and also in the lateral line^28^. In mammals, mature BDNF can bind to two TrkB receptor isoforms: full-length TrkB and truncated TrkB. While the full-length TrkB undergoes autophosphorylation to activate intracellular signaling pathways in response to BDNF binding, the truncated TrkB lacks the intracellular tyrosine kinase domain and thus cannot undergo autophosphorylation. Instead, this TrkB isoform acts to internalize BDNF, serving as a dominant-negative receptor that indirectly inhibits BDNF function^15^. However, another study demonstrated the truncated TrkB as an active form^29^. TrkB is almost exclusively expressed in the brain in mammals^30^; however, it has a wider distribution in fish, including the brain^3,5,14^, gut^31^, kidney^32^ and the lateral line system^20^. Besides TrkB, mammalian BDNF can bind to the p75 neurotrophin receptor (p75NTR), although with low-affinity^33^. While this receptor is present in fish^15,25^, its distribution and mechanisms of actions in this vertebrate group is yet to be explored. TrkB and p75NTR seem to be involved in different actions of BDNF, at least in mammals. Thus, TrkB receptors are proposed to mediate neuronal survival, spine formation and maturation, whereas p75NTR may be involved in a variety of actions ranging from trophism to apoptosis^34,35^.

BDNF is well known for promoting the differentiation of neurons from stem cells, enhancing synaptogenesis, controlling the synaptic interactions that influence memory and cognition mechanisms, and preventing apoptosis in the brain of mammals [see reviews^36–38^]. Additionally, BDNF has been associated with neurogenesis and neurogeneration in the mammalian brain, although results on this topic are controversial^15^. In fish, studies on the BDNF actions in the brain are scarce, but it has been recently demonstrated that BDNF contributes to the establishment of new neuronal population in the damaged brain of zebrafish^39^. Besides the neurotrophic properties of BDNF, emerging findings in mammals have described an important role for this peptide in the regulation of feeding and energy balance [reviewed in^40–42^]. Central administration of BDNF suppresses appetite and induces a decrease in body weight in rodents^36–39^. Accordingly, different BDNF- and TrkB-transgenic rodent models show increased body weight and food intake levels, accompanied by hyperglycemia, hyperinsulinemia and hyperleptinemia^40–44^. Effects of BDNF on energy balance are mainly characterized by a hypoglycemic action. Thus, BDNF lowers blood glucose levels when administered to diabetic mice^45,46^, an action that seems to be associated with the capacity of BDNF to increase the number of pancreatic islets and secretory granules in β-cells^46^. Additionally, infusion of BDNF into the brain decreases glucagon levels in the portal vein of rats^47^. BDNF has also been shown to reduce serum levels of non-esterified free fatty acids, total cholesterol and phospholipids, as well as the liver triglyceride content in diabetic mice^48^, which points to a role for the peptide in lipid metabolism as well.

To the best of our knowledge, no reports are available to date on a putative role for BDNF on feeding and energy balance regulation in fish. Additionally, the peripheral distribution of BDNF in fish is poorly known. This research aims to study the tissue distribution of the BDNF system, its regulation by feeding and nutritional status, and its putative role in food intake and glucose and lipid metabolism in zebrafish (*Danio rerio*). Specifically, our objectives were to: (1) describe the tissue distribution of BDNF and its receptors (TrkB2, p75NTRA and p75NTRB) in zebrafish, (2) determine the periprandial and fasting-induced expression of the BDNF system (peptide and its above-mentioned receptors) in zebrafish central and peripheral tissues, (3) study the action of BDNF on zebrafish food intake, (4) study the putative role of BDNF on the expression and activity of enzymes related to glucose and lipid metabolism in zebrafish liver in vivo and in vitro, and (5) determine whether the coactivator peroxisome proliferator-activated receptor gamma coactivator 1-alpha (Ppargc1α) and the transcription factor peroxisome proliferator-activated receptor alpha (Pparα) (both known to mediate BDNF actions in mammals) may also operate in zebrafish. Our results show that BDNF is a meal-responsive orexigen in zebrafish. This report offers the first body of evidence linking BDNF to appetite and energy balance regulation in a non-mammal.

## Results

### The BDNF system is widely distributed within the zebrafish tissues

**Figure 1:**
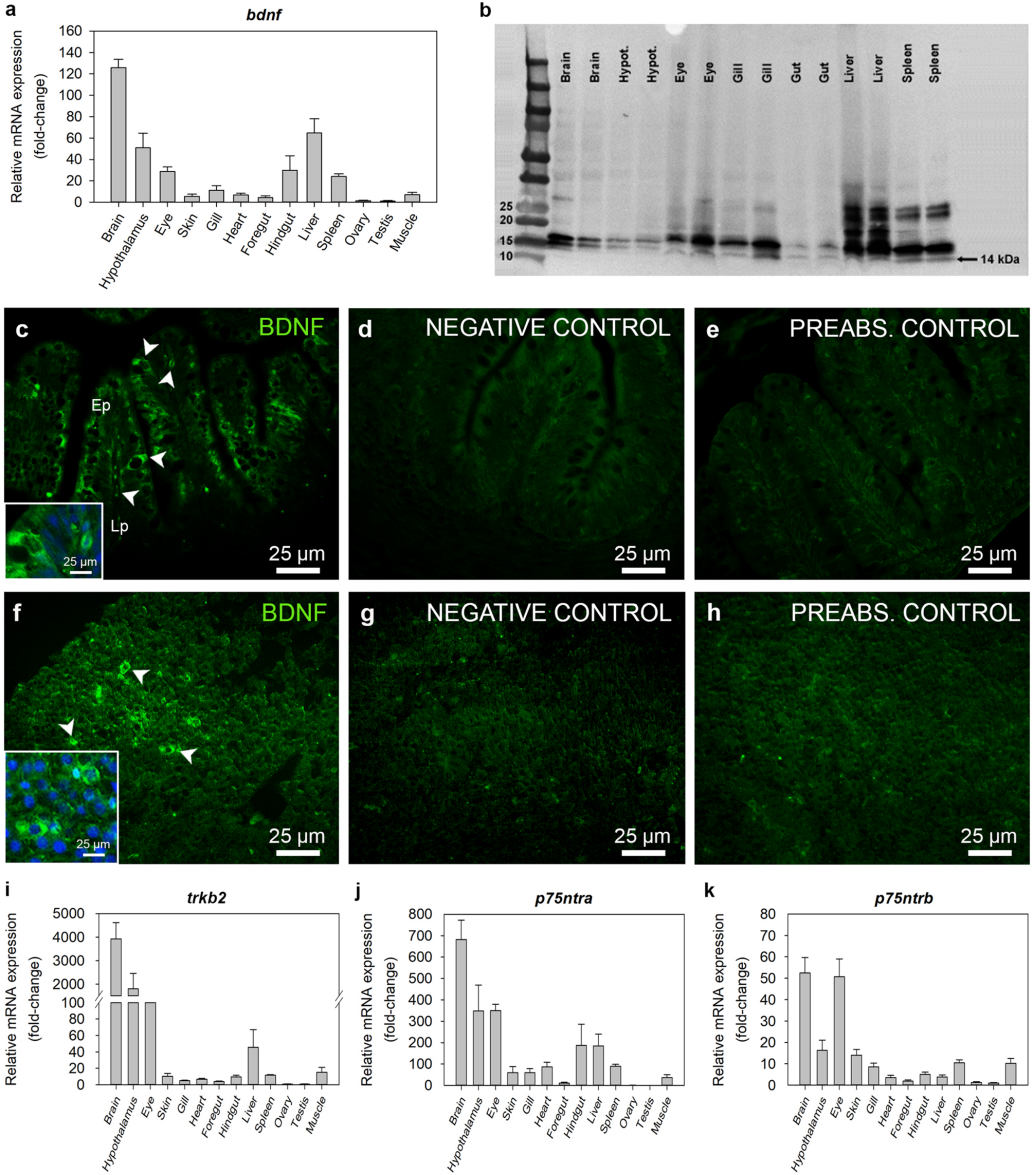
Distribution of BDNF and its receptors in the zebrafish. (**a**) Tissue distribution of *bdnf* mRNAs in zebrafish. Quantitative analysis of mRNA expression was performed by RT-qPCR considering *β-actin* as reference gene. Data are expressed as mean + SEM (n = 6), relative to the tissue with the lowest mRNA expression. (**b**) Full-length Western blot image showing BDNF protein in zebrafish tissues (n = 2). Protein molecular weight (in kDa) is shown in figure. (**c**–**h**) Representative sections of zebrafish gut (**c**–**e**) and liver (**f**–**h**) showing BDNF immunofluorescence (green). A magnified image of representative cells immunopositive for BDNF is shown in a square inset for both foregut and liver. In insets, nuclei are stained blue (DAPI). No or small immunoreactivity was detected in negative (**d**,**g**) or preabsorption (**e**,**h**) controls. Scale bars are indicated in each image. (**i**–**k**) Tissue distribution of mRNAs encoding BDNF receptors in zebrafish. Data obtained by RT-qPCR are expressed as mean + SEM (n = 6), relative to the tissue with the lowest mRNA expression. *Ac* absorptive cell, *BDNF* brain-derived neurotrophic factor, *Ep* epithelium, *Lp* lamina propria, *p75ntr* neurotrophin receptor p75, *trkb* tropomyosin receptor kinase B.

Abundance of *bdnf* mRNAs was detected in several tissues of the zebrafish. The tissue with the highest expression was the whole brain (without the hypothalamus), followed by the hypothalamus and liver. Lesser, but significant levels of *bdnf* mRNAs were also found in the eye, hindgut and spleen, while a minimum expression was detected in the skin, gill, heart, foregut and muscle. Expression of the *bdnf* mRNA was almost undetectable in the ovary and testis (Fig. 1a). Western blot analysis detected the processed, mature form of BDNF (14 kDa) in the zebrafish brain (without the hypothalamus), hypothalamus, eye, gill, gut, liver and spleen. Additional bands were also observed in blots, which may correspond to different isoforms and/or glycosylated forms of BDNF and/or pro-BDNF (Fig. 1b). Additionally, BNDF-like immunoreactivity was found in the zebrafish foregut (Fig. 1c) and liver (Fig. 1f). BDNF-like signal within the gut was observed along the epithelium and in the lamina propria. In the liver, BDNF-like immunoreactivity was detected in scattered cells surrounding the nucleus. No or small signal was detected in gut and liver sections stained with secondary antibody alone (Fig. 1d,g) and in the preabsorption controls (Fig. 1e,h).

BDNF receptors (TrkB2, p75NTRa and p75NTRb) were mainly expressed in the brain and in the eye of zebrafish, although they were also detected in some peripheral tissues (Fig. 1i–k). Specifically, *tkrb2* mRNAs within the periphery were almost restricted to the liver, although low levels were also detected in the skin, heart, hindgut, spleen and muscle (Fig. 1i). A considerable amount of mRNAs encoding p75NTRa was detected in the hindgut and liver, followed by the skin, gill, heart, spleen and muscle (Fig. 1j). Expression of both *trkb2* and *p75ntra* mRNAs was almost undetectable, and therefore unquantifiable, in foregut and gonads (both ovary and testis) (Fig. 1i,j). Finally, *p75ntrb* mRNAs were mainly found in the skin, gill, spleen and muscle, with lower levels in the heart, foregut, hindgut and liver (Fig. 1k).

The BDNF system is expressed at very low levels in the zebrafish foregut (Fig. 1), and the mRNAs encoding TrkB2 and p75NTRA were unquantifiable and the Ct values were about 35. Hence, we did not measure these mRNAs in the foregut in subsequent experiments. However, although low, detectable levels of *bdnf* and *p75ntrb* were found in the foregut. Therefore, we continued to investigate them in the foregut, given its importance in food intake and energy balance regulation.

### Preprandial and postprandial expression of the BDNF system in the zebrafish brain, liver and foregut

**Figure 2:**
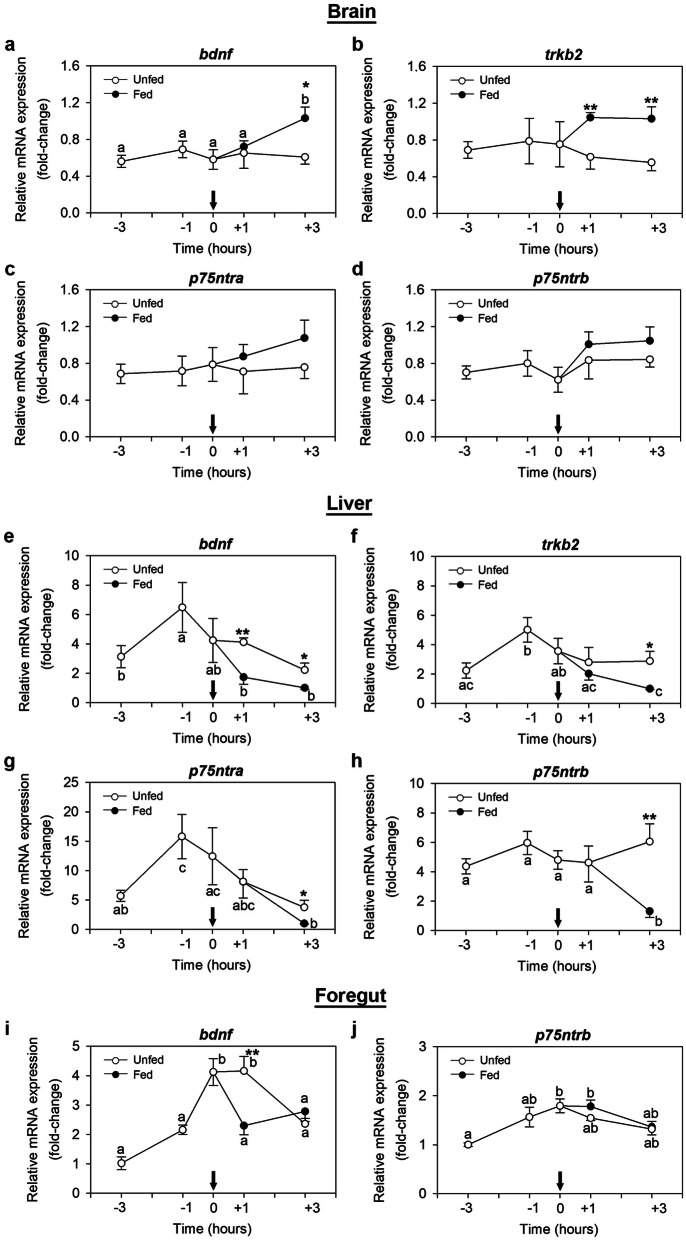
Periprandial changes in the levels of mRNAs encoding BDNF and its receptors in the zebrafish brain (**a**–**d**), liver (**e**–**h**) and foregut (**i**,**j**). Samples were collected before scheduled feeding time (− 3 h and − 1 h), at feeding time (0 h) and after scheduled feeding time (+ 1 h and + 3 h) in both fed and unfed fish. Data are expressed as mean ± SEM (n = 6) relative to the lowest average expression. Arrows denote feeding time. Different letters indicate significant differences (p < 0.05) among the different time points in fed (black dots) or unfed (white dots) groups, while asterisks indicate significant differences (*p < 0.05, **p < 0.01) between groups at the same time point. *Bdnf *brain-derived neurotrophic factor, *p75ntr* neurotrophin receptor p75, *trkb* tropomyosin receptor kinase B.

The periprandial variations in the expression of *bdnf*, *trkb2*, *p75ntra* and *p75ntrb* mRNAs in the zebrafish brain, liver and foregut are shown in Fig. 2. In the brain, *bdnf* mRNAs were observed to rise significantly at 3 h after feeding in those fish who received food at their scheduled feeding time [ANOVA significance values: *bdnf*: F (4, 25) = 4.119, p = 0.011] (Fig. 2a). Levels of *trkb2* transcript in fed fish were not significantly different among time points; however, they were considerably higher than those of unfed fish at 1 and 2 h post-scheduled feeding (p = 0.006 and 0.007, respectively) (Fig. 2b). No periprandial variations were detected in the expression of *p75ntra* and *p75ntrb* in the brain (Fig. 2c,d).

In the liver, a significant preprandial increase in *bdnf*, *trkb2* and *p75ntra* mRNA levels was observed at 1 h before the scheduled feeding (− 1 h) compared to − 3 h values [ANOVA significance values: *bdnf*: F (4, 25) = 2.912, p = 0.042; *trkb2*: F (4, 25) = 6.218, p = 0.001; *p75ntra*: F (4, 25) = 3.597, p = 0.019]. For these three genes, feeding decreased mRNA up to levels similar or slightly lower than its expression at 3 h before the regular feeding time. Such postprandial mRNA levels in fed fish were, in all cases, significantly lower than in fish that missed their scheduled feeding (*bdnf*: p = 0.002 and 0.029; *trkb2*: p = 0.017; *p75ntra*: p = 0.023) (Fig. 2e–g). Expression levels of *p75ntrb* in the liver were significantly lower at 3 h after the scheduled feeding compared to the rest of the values [ANOVA significance values: F (4, 25) = 4.685, p = 0.006], which remained unaltered in both fed and unfed fish (Fig. 2h).

In the foregut, *bdnf* mRNAs increased significantly during – 3 to − 1 h to the scheduled feeding time, and drastically decreased at 1 h post-feeding in fed fish [ANOVA significance values: F (4, 25) = 12.690, p ≤ 0.001]. Meanwhile, the levels of *bdnf* remained high at + 1 h in those fish that missed the scheduled feeding, although a significant drop was detected at + 3 h in this group [ANOVA significance values: F (4, 25) = 16.458, p ≤ 0.001] (Fig. 2i). Expression of *p75ntrb* also rose preprandially in the foregut. However, no significant postprandial variations were detected neither in fed nor unfed fish when compared to values at scheduled feeding time, and the values at + 3 h returned to levels seen at − 3 h [ANOVA significance values: fed fish: F (4, 25) = 6.038, p = 0.002; unfed fish: F (4, 25) = 5.680, p = 0.002] (Fig. 2j).

### Food availability is an important modulator of the expression of BDNF and its receptors in zebrafish

**Figure 3:**
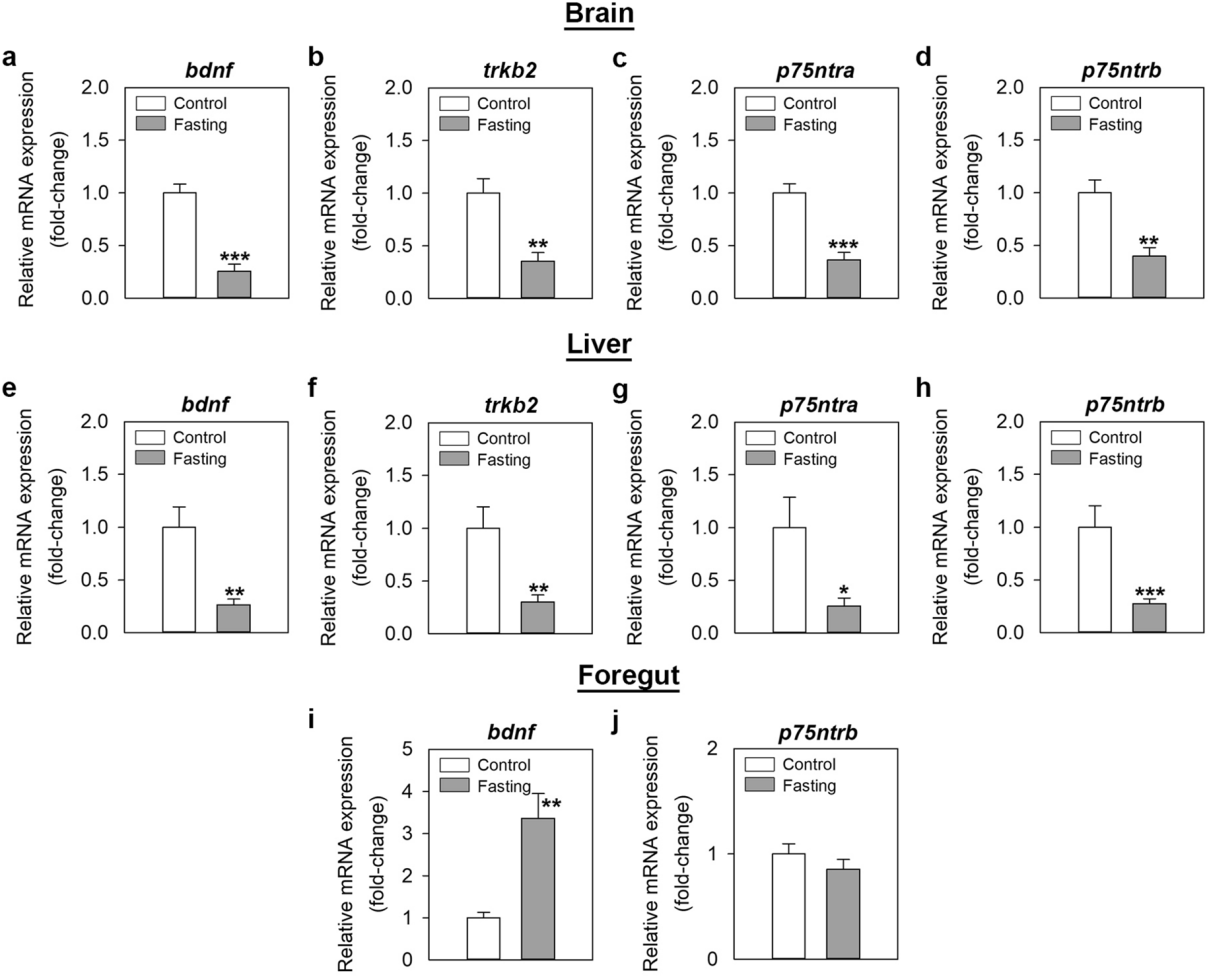
Effects of 7-day fasting on the mRNA expression of *bdnf* and its receptors in the zebrafish brain (**a**–**d**), liver (**e**–**h**) and foregut (**i**,**j**). Data obtained by RT-qPCR are expressed as mean + SEM (n = 6). Asterisks denote significant differences between control and treated groups assessed by t-test (*p < 0.05, **p < 0.01, ***p < 0.001). *bdnf* brain-derived neurotrophic factor, *p75ntr* neurotrophin receptor p75, *trkb* tropomyosin receptor kinase B.

Food deprivation for 7 days significantly downregulated the expression of *bdnf* (≈ 3-fold, p ≤ 0.001), *trkb2* (≈ 3-fold; p = 0.002), *p75ntra* (≈ 3-fold; p ≤ 0.001) and *p75ntrb* (≈ 2.5-fold; p = 0.002) mRNAs in the zebrafish brain (Fig. 3a–d). Similar decreases in the expression of all genes [*bdnf*: ≈ 4-fold; p = 0.003; *trkb2*: ≈ 3-fold, p = 0.009; *p75ntra*: (≈ 4-fold), p = 0.031; *p75ntrb*: ≈ 3.5-fold, p ≤ 0.001] were observed in the liver of fasted fish (Fig. 3e–h). On the contrary, *bdnf* mRNA expression was significantly higher (≈ 3.5-fold) in the foregut of 7-day food-deprived fish when compared to the control fed fish (p = 0.005) (Fig. 3i). Foregut levels of *p75ntrb* mRNA remained unaltered by a 7-day fasting period (Fig. 3j).

### BDNF is an orexigen in zebrafish

**Figure 4:**
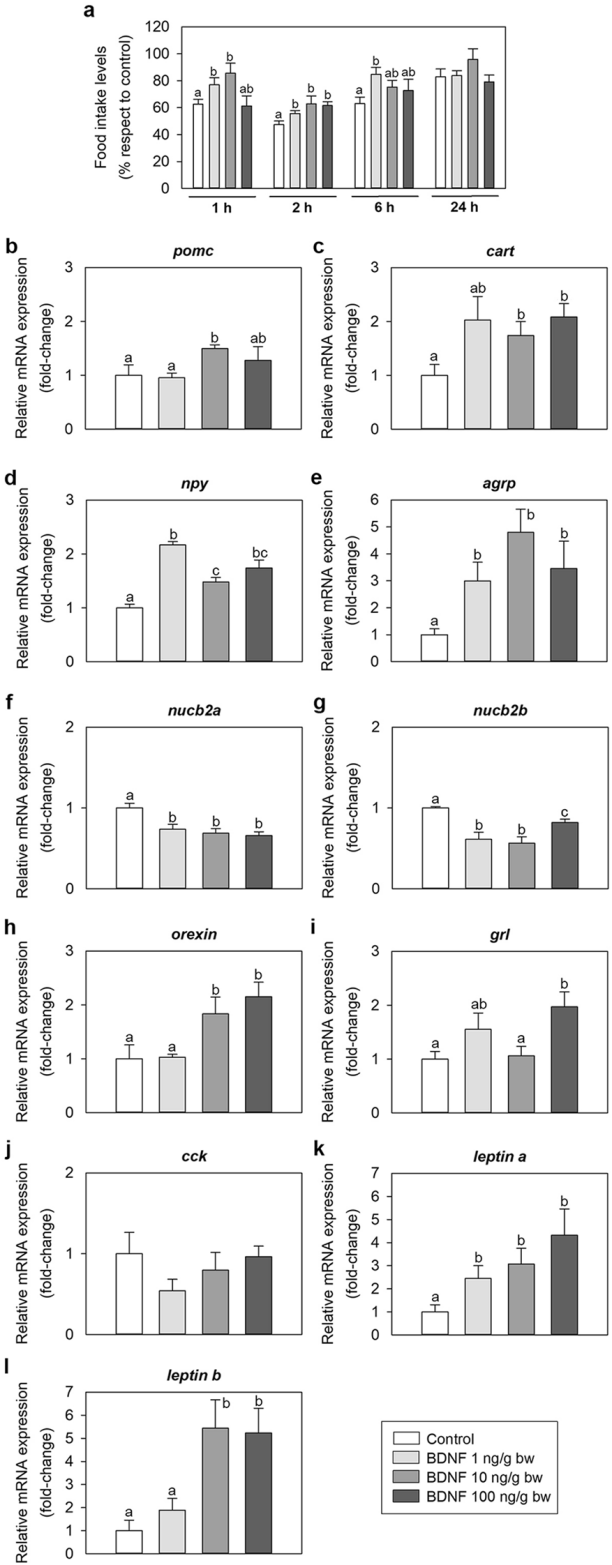
Effects of BDNF on feeding regulation in zebrafish. (**a**) Food intake 1, 2, 6 and 24 h after the intraperitoneal administration of saline alone (control) or containing 1, 10 or 100 ng/g bw of BDNF. Levels of food intake are represented as mean + SEM of the percentage of food ingested with respect to baseline levels (calculated as the average of food intake the 3 days previous to experiment). Results correspond to the mean + SEM of the results obtained in three different experiments (n = 3 in each experiment). Different letters indicate significant differences (p < 0.05) among groups assessed by one-way ANOVA and SNK test. (**b**–**l**) Expression of mRNAs encoding key appetite-regulating peptides in the zebrafish brain (**b**–**h**), foregut (**i**–**j**) and liver (**k**–**l**) 2 h after intraperitoneal administration of saline alone (control) or containing 1, 10 or 100 ng/g bw of BDNF. Data obtained by RT-qPCR are expressed as mean + SEM (n = 6). Different letters indicate significant differences (p < 0.05) among groups assessed by one-way ANOVA and SNK test. *Agrp *agouti-related protein, *BDNF* brain-derived neurotrophic factor, *cart* cocaine- and amphetamine-regulated transcript, *cck* cholecystokinin, *grl* ghrelin, *npy* neuropeptide Y, *nucb2* nucleobindin 2, *pomc* proopiomelanocortin.

BDNF increased food intake of zebrafish when administered intraperitoneally. Increases were found to be significant at 1 h [with 1 and 10 ng/g bw BDNF doses; ANOVA significance values: F (3, 20) = 3.603, p = 0.031; Student–Newman–Keuls (SNK) values: p = 0.044 and 0.025, respectively], 2 h [all doses tested; ANOVA significance values: F (3, 20) = 3.737, p = 0.028; SNK: p = 0.040, 0.037 and 0.01, respectively] and 6 h [only with 1 ng/g bw dose; ANOVA significance values: F (3, 20) = 3.149, p = 0.048; SNK: p = 0.020] post-injection. There were no significant differences in food intake between control and treated groups at 24 h post-injection (Fig. 4a). IP administration of all doses of BDNF also caused a significant increase in *neuropeptide y* [*npy*; ANOVA: F (3, 20) = 25.413, p < 0.001; SNK: p < 0.001 for all doses] and *agouti-related protein* [*agrp*; ANOVA: F (3, 20) = 4.344, p = 0.016; SNK: p = 0.045, 0.01 and 0.047, respectively] mRNAs, and a significant reduction in *nucleobindin 2a* [*nucb2a*; ANOVA: F (3, 20) = 7.571, p = 0.001; SNK: p = 0.004, 0.003 and 0.002] and *nucleobindin 2b* [*nucb2b*; ANOVA: F (3, 20) = 10.151, p < 0.001; SNK: p ≤ 0.001, < 0.001 and 0.045] in the zebrafish brain at 2 h post-injection (Fig. 4d–g). There was also a significant upregulation of *proopiomelanocortin* (*pomc*) mRNA expression in the brain upon the injection of 10 ng/g bw BDNF [ANOVA: F (3, 20) = 3.235, p = 0.044; SNK: p = 0.045] (Fig. 4b), and of *cocaine- and amphetamine-regulated transcript* (*cart*) and *orexin* mRNA expression after the injection of 10 and 100 ng/g bw BDNF [*cart*: ANOVA: F (3, 20) = 3.511, p = 0.041; SNK: p = 0.049 and 0.042; *orexin*: ANOVA: F (3, 20) = 5.591, p = 0.006; SNK: p = 0.043 and 0.017] (Fig. 4c,h). In the foregut, BDNF significantly induced *ghrelin* (*grl*) mRNA levels when IP injected at a dose of 100 ng/g bw [ANOVA: F (3, 20) = 3.771, p = 0.027; SNK: p = 0.039], although no changes were observed at lower doses (Fig. 4i). Levels of *cholecystokinin* (*cck*) mRNA was unaltered by BDNF injection in the foregut (Fig. 4j). Finally, a significant increase in *leptin a* and *leptin b* was detected in the liver of BDNF-injected fish when compared to saline-injected fish. Such an increase in expression was statistically significant after injection of all doses of BDNF for *leptin a* [ANOVA: F (3, 20) = 3.544, p = 0.0.33; SNK: p = 0.049, 0.047 and 0.021, respectively], and with 10 and 100 ng/g bw BDNF for *leptin b* [ANOVA: F (3, 20) = 6.610, p = 0.003; SNK: p = 0.01 and 0.008] (Fig. 4k,l).

### BDNF modulates the hepatic expression of genes involved in glucose and lipid metabolism in vivo and in vitro

Table 1 shows the abundance of genes (mRNAs) involved in glucose and lipid metabolism in response to IP administration of BDNF. All doses of peptide tested (1, 10 and 100 ng/g bw) caused a significant increase in *fructose 1,6-bisphosphatase 1a* [*fbp1a*; ANOVA: F (3, 20) = 3.635, p = 0.031; SNK: p = 0.033, 0.045 and 0.037, respectively], *glucose 6-phosphatase b* [*g6pcb*; ANOVA: F (3, 20) = 11.304, p < 0.001; SNK: p = 0.042, 0.028 and < 0.001], *glycogen phosphorylase* [ANOVA: F (3, 20) = 8.450, p < 0.001; SNK: p = 0.049, 0.002 and 0.002], *ATP citrate lyase* [*acly*; ANOVA: F (3, 20) = 3.972, p = 0.023; SNK: p = 0.016, 0.045 and 0.043], *acetyl-CoA carboxylase* [*acaca*; ANOVA: F (3, 20) = 5.257, p = 0.008; SNK: p = 0.045, 0.048 and 0.007], *carnitine palmitoyltransferase 1a* [*cpt1a*; ANOVA: F (3, 20) = 7.515, p = 0.001; SNK: p = 0.048, 0.003 and 0.003], *3-hydroxy-3-methylglutaryl-CoA lyase* [*hmgcl*; ANOVA: F (3, 20) = 5.844, p = 0.005; SNK: p = 0.002, 0.048 and 0.042] and *acetyl-CoA acetyltransferase 1* [*acat1*; ANOVA: F (3, 20) = 5.865, p = 0.024; SNK: p = 0.015, 0.031 and 0.042] mRNAs at 2 h post-injection when compared to saline-treated fish. Levels of *phosphofructokinase a* [*pfkla*; ANOVA: F (3, 20) = 8.055, p = 0.001; SNK: p = 0.007], *phosphofructokinase b* [*pfklb*; ANOVA: F (3, 20) = 8.610, p < 0.001; SNK: p = 0.034 and 0.002], *phosphoenolpyruvate carboxykinase 2* [*pck2*; ANOVA: F (3, 20) = 5.829, p = 0.005; SNK: p = 0.048 and 0.012], *fatty acid synthase* [*fasn*; ANOVA: F (3, 20) = 3.381, p = 0.038; SNK: p = 0.029] and *3-hydroxyacyl CoA dehydrogenase* [*hadh*; ANOVA: F (3, 20) = 10.457, p < 0.001; SNK: p < 0.001] were also upregulated by BDNF but in a dose-dependent manner. There was also a dose-dependent decrease in the levels of *pyruvate kinase* [*pklr*; ANOVA: F (3, 20) = 7.421, p = 0.002; SNK: p = 0.016 and < 0.001], *fructose 1,6-bisphosphatase 1b* [*fbp1b*; ANOVA: F (3, 20) = 4.321, p = 0.017; SNK: p = 0.024], *acyl-CoA dehydrogenase medum-chain* [*acadm*; ANOVA: F (3, 20) = 4.191, p = 0.019; SNK: p = 0.012] and *enoyl-CoA hydratase short-chain 1* [*echs1*; ANOVA: F (3, 20) = 6.511, p = 0.003; SNK: p = 0.004] in response to IP BDNF. Expression of *glucose transporter 2* (*glut2*), *sodium-glucose cotransporter 1* (*sglt1*), *glucokinase* (*gck*) and *phosphoenolpyruvate carboxykinase 1* (*pck1*) remained unaltered upon treatment with BDNF. Apart from the genes related to glucose and lipid metabolism, the expression of key transcription factors (*ppargc1α* and *pparα*) was measured. Levels of both these genes were observed to be upregulated by the administration of 100 ng/g bw BDNF [*ppargc1α*: ANOVA: F (3, 20) = 9.394, p < 0.001; SNK: p = 0.006; *pparα*: ANOVA: F (3, 20) = 5.307, p = 0.007; SNK: 0.047] (Table 1).

**Table 1:** Effects of intraperitoneal administration of BDNF on the expression of genes involved in glucose and lipid transport and metabolism and of key transcription factors in the zebrafish liver.

	Control	BDNF 1 ng/g bw	BDNF 10 ng/g bw	BDNF 100 ng/g bw
**Glucose transport and metabolism**				
Transport:				
* glut2*	1.00 ± 0.27	0.83 ± 0.28	0.72 ± 0.14	1.32 ± 0.27
* sglt1*	1.00 ± 0.33	0.49 ± 0.10	0.51 ± 0.10	1.28 ± 0.35
Glycolysis:				
* gck*	1.00 ± 0.19	0.90 ± 0.31	0.76 ± 0.30	0.90 ± 0.39
* pfkla*	1.00 ± 0.15a	0.47 ± 0.15a	0.94 ± 0.09a	1.92 ± 0.36b
* pfklb*	1.00 ± 0.16a	0.85 ± 0.28a	2.34 ± 0.55b	3.45 ± 0.54b
* pklr*	1.00 ± 0.12a	0.54 ± 0.08b	0.31 ± 0.06b	0.64 ± 0.14ab
Gluconeogenesis:				
* pck1*	1.00 ± 0.10	1.05 ± 0.11	0.90 ± 0.12	0.89 ± 0.05
* pck2*	1.00 ± 0.07a	1.77 ± 0.21b	1.33 ± 0.32ab	2.09 ± 0.49b
* fbp1a*	1.00 ± 0.25a	2.98 ± 0.56b	2.14 ± 0.24b	2.76 ± 0.66b
* fbp1b*	1.00 ± 0.18a	1.03 ± 0.15a	0.35 ± 0.05b	1.26 ± 0.29a
* g6pcb*	1.00 ± 0.17a	5.80 ± 0.94b	5.69 ± 0.60b	12.46 ± 2.56c
Glycogenolysis:				
* glycogen phosphorylase*	1.00 ± 0.12a	2.35 ± 0.58b	4.52 ± 0.89b	4.31 ± 0.43b
Lipid metabolism				
Fatty acid synthesis:				
* acly*	1.00 ± 0.18a	27.06 ± 9.20b	10.19 ± 2.23b	17.42 ± 5.76b
* acaca*	1.00 ± 0.18a	3.06 ± 0.66bc	1.85 ± 0.18ab	4.41 ± 1.09c
* fasn*	1.00 ± 0.32a	2.09 ± 0.51ab	1.66 ± 0.32ab	2.61 ± 0.54b
Β-oxidation:				
* cpt1a*	1.00 ± 0.44a	7.04 ± 2.50b	16.25 ± 4.19b	17.25 ± 2.84b
* acadm*	1.00 ± 0.22a	0.75 ± 0.13ab	0.42 ± 0.13b	0.81 ± 0.10ab
* echs1*	1.00 ± 0.22a	0.93 ± 0.12a	0.25 ± 0.07b	0.63 ± 0.08a
* hadh*	1.00 ± 0.34a	2.30 ± 0.19b	0.71 ± 0.07a	0.96 ± 0.20a
Ketogenesis:				
* hmgcl*	1.00 ± 0.36a	4.62 ± 0.78b	2.76 ± 0.47b	2.74 ± 0.73b
* acat1*	1.00 ± 0.15a	2.39 ± 0.36b	2.45 ± 0.51b	2.47 ± 0.61b
**Transcriptional regulators**				
*ppargc1α*	1.00 ± 0.45a	0.33 ± 0.06a	1.08 ± 0.26a	2.46 ± 0.26b
*pparα*	1.00 ± 0.20a	1.30 ± 0.21ab	0.62 ± 0.10a	1.52 ± 0.14b

Fish were ip injected with saline alone (control) or containing 1, 10 or 100 ng/g bw of BDNF and samples were collected 1 h post-injection. Data obtained by RT-qPCR are expressed as mean + SEM (n = 6). Different letters indicate significant differences (p < 0.05) among groups assessed by one-way ANOVA and SNK test.

*acaca*, *acetyl-CoA carboxylase; acadm*, *acyl-CoA dehydrogenase medium-chain*; *acat1*, *acetyl-CoA acetyltransferase 1*; *acly*, *ATP citrate lyase*; BDNF, brain-derived neurotrophic factor; *cpt1a*, *carnitine palmitoyltransferase 1a*; *echs1*, *enoyl-CoA hydratase short-chain 1*; *fasn*, *fatty acid synthase*; *fbp1a*, *fructose 1,6-bisphosphatase 1a*; *fbp1b*, *fructose 1,6-bisphosphatase 1b*; *g6pcb*, *glucose 6-phosphatase b*; *gck*, *glucokinase*; *glut2*, *glucose transporter 2*; *hadh*, *3-hydroxyacyl CoA dehydrogenase*; *hmgcl*, *3-hydroxy-3-methylglutaryl-CoA lyase*; *pck1*, *phosphoenolpyruvate carboxykinase 1*; *pck2*, *phosphoenolpyruvate carboxykinase 2*; *pfkla*, *phosphofructokinase a*; *pfklb*, *phosphofructokinase b*; *pklr*, *pyruvate kinase*; *pparα*, *peroxisome proliferator-activated receptor alpha*; *ppargc1α*, *peroxisome proliferator-activated receptor gamma coactivator 1-alpha*; *sglt1*, *sodium-glucose cotransporter 1.*

The effects of in vitro treatment of ZFL cells with BDNF on the expression of genes involved in glucose and lipid metabolism, mitochondrial activity and transcription factors are shown in Table 2. Almost all of the genes studied (*glut2*, *sglt1*, *gck*, *pklr*, *pck2*, *fbp1a*, *g6pcb*, *glycogen phosphorylase*, *acly*, *acaca*, *fasn*, *cpt1a*, *hmgcl*, *acat1*, *ppargc1α* and *pparα*) were significantly upregulated in response to exposure of cells to 0.1, 1 and/or 10 nM BDNF during 1 and 6 h [significance values: *glut2*: 1 h—ANOVA: F (3, 20) = 11.050, p < 0.001; SNK: p = 0.049 and < 0.001; 6 h—ANOVA: F (3, 20) = 5.419, p = 0.007; SNK: p = 0.045 and 0.004; *sglt1*: 1 h—ANOVA: F (3, 20) = 25.376, p < 0.001; SNK: p = 0.008, < 0.001 and < 0.001; 6 h—ANOVA: F (3, 20) = 4.572, p = 0.014; SNK: p = 0.032, 0.038 and 0.009; *gck*: 1 h—ANOVA: F (3, 20) = 10.981, p < 0.001; SNK: p = 0.048, 0.036 and < 0.001; 6 h—ANOVA: F (3, 20) = 3.115, p = 0.049; SNK: p = 0.039, 0.045 and 0.035; *pklr*: 1 h—ANOVA: F (3, 20) = 46.488, p < 0.001; SNK: p = 0.016 and < 0.001; 6 h—ANOVA: F (3, 20) = 10.503, p < 0.001; SNK: p = 0.004, 0.024 and < 0.001; *pck2*: 1 h—ANOVA: F (3, 20) = 18.407, p < 0.001; SNK: p ≤ 0.001 for all doses; 6 h—ANOVA: F (3, 20) = 10.399, p < 0.001; SNK: p ≤ 0.001, = 0.006 and < 0.001; *fbp1a*: 1 h—ANOVA: F (3, 20) = 11.082, p < 0.001; SNK: p = 0.045 and < 0.001; 6 h—ANOVA: F (3, 20) = 9.953, p < 0.001; SNK: p = 0.004, 0.002 and < 0.001; *g6pcb*: 1 h—ANOVA: F (3, 20) = 12.007, p < 0.001; SNK: p = 0.046, 0.035 and < 0.001; 6 h—ANOVA: F (3, 20) = 11.557, p < 0.001; SNK: p ≤ 0.001, = 0.068 and < 0.001; *glycogen phosphorylase*: 1 h—ANOVA: F (3, 20) = 8.058, p = 0.001; SNK: p = 0.016, 0.004 and < 0.001; 6 h—ANOVA: F (3, 20) = 10.825, p < 0.001; SNK: p = 0.025, 0.02 and < 0.001; *acly*: 1 h—ANOVA: F (3, 20) = 35.279, p < 0.001; SNK: p ≤ 0.001 and < 0.001; 6 h—ANOVA: F (3, 20) = 22.776, p < 0.001; SNK: p = 0.048 and < 0.001; *acaca*: 1 h—ANOVA: F (3, 20) = 17.978, p < 0.001; SNK: p ≤ 0.001 and = 0.006; 6 h—ANOVA: F (3, 20) = 4.738, p = 0.012; SNK: p = 0.05 and 0.027; *fasn*: 1 h—ANOVA: F (3, 20) = 5.364, p = 0.026; SNK: p = 0.019, 0.043 and 0.030; 6 h—ANOVA: F (3, 20) = 5.970, p = 0.004; SNK: p = 0.041, 0.007 and 0.005; *cpt1a*: 1 h—ANOVA: F (3, 20) = 9.359, p < 0.001; SNK: p = 0.005 and 0.002; 6 h—ANOVA: F (3, 20) = 9.144, p < 0.001; SNK: p = 0.009 and 0.005; *hmgcl*; 1 h—ANOVA: F (3, 20) = 9.458, p < 0.001; SNK: p = 0.006 and 0.001; 6 h—ANOVA: F (3, 20) = 9.023, p < 0.001; SNK: p = 0.046 and < 0.001; *acat1*: 1 h—ANOVA: F (3, 20) = 17.580, p < 0.001; SNK: p = 0.05 and < 0.001; 6 h—ANOVA: F (3, 20) = 13.829, p < 0.001; SNK: p = 0.01 and < 0.001; *ppargc1α*: 1 h—ANOVA: F (3, 20) = 9.389, p < 0.001; SNK: p = 0.011 and < 0.001; 6 h—ANOVA: F (3, 20) = 6.089, p = 0.004; SNK: p = 0.003, 0.007 and 0.008; *pparα*: 1 h—ANOVA: F (3, 20) = 13.615, p < 0.001; SNK: p = 0.046, < 0.001 and < 0.001; 6 h—ANOVA: F (3, 20) = 6.667, p = 0.003; SNK: p = 0.008, 0.028 and 0.002]. Expression of *fbp1b* was also increased by BDNF but only at 1 h [ANOVA: F (3, 20) = 6.567, p = 0.003; SNK: p = 0.012]. Genes that were most significantly induced include *glut2*, *sglt1*, *pklr* and *hmgcl* (magnitude of induction of ≈ 6 to 7 fold in response to the highest BDNF concentration), while the smallest magnitudes of induction were detected for *pck2*, *cpt1a* and *pparα* (≈ 2 to 3 fold). In vitro treatment with BDNF also caused a significant reduction in the mRNA levels of *acadm* [1 h—ANOVA: F (3, 20) = 3.710, p = 0.029; SNK: p = 0.045, 0.022 and 0.045; 6 h—ANOVA: F (3, 20) = 4.528, p = 0.014; SNK: p = 0.015), *echs1* (1 h—ANOVA: F (3, 20) = 4.095, p = 0.020; SNK: p = 0.021 and 0.040; 6 h—ANOVA: F (3, 20) = 4.652, p = 0.013; SNK: p = 0.049 and 0.007) and *hadh* (1 h—ANOVA: F (3, 20) = 7.769, p = 0.001; SNK: p = 0.01 and 0.008; 6 h—ANOVA: F (3, 20) = 4.060, p = 0.021; SNK: p = 0.013] at 1 and 6 h post-incubation. No changes in mRNA expression in response to BDNF exposure were detected for *pfkla*, *pfklb* and *pck1*.

**Table 2:** In vitro effects of the exposure to BDNF on the expression of genes involved in glucose and lipid transport and metabolism and of key transcription factors in ZFL cells.

	1 h				6 h			
	Control	BDNF 0.1 nM	BDNF 1 nM	BDNF 10 nM	Control	BDNF 0.1 nM	BDNF 1 nM	BDNF 10 nM
**Glucose transport and metabolism**								
Transport:								
* glut2*	1.00 ± 0.11a	1.52 ± 0.59ab	2.55 ± 0.24b	5.07 ± 0.84c	1.00 ± 0.20a	1.53 ± 0.23ab	1.75 ± 0.30b	2.28 ± 0.15b
* sglt1*	1.00 ± 0.24a	2.82 ± 0.03b	3.92 ± 0.74bc	5.91 ± 0.39c	1.00 ± 0.08a	1.90 ± 0.09b	1.95 ± 0.35b	2.42 ± 0.41b
Glycolysis:								
* gck*	1.00 ± 0.11a	1.46 ± 0.06b	2.02 ± 0.25b	3.48 ± 0.59c	1.00 ± 0.09a	1.79 ± 0.10b	1.85 ± 0.12b	2.24 ± 0.56b
* pfkla*	1.00 ± 0.11	1.00 ± 0.15	1.08 ± 0.08	0.71 ± 0.31	1.00 ± 0.10	1.10 ± 0.19	1.38 ± 0.38	0.81 ± 0.21
* pfklb*	1.00 ± 0.15	1.63 ± 0.05	1.11 ± 0.13	0.98 ± 0.38	1.00 ± 0.06	1.03 ± 0.23	0.99 ± 0.24	1.12 ± 0.17
* pklr*	1.00 ± 0.25a	1.52 ± 0.20ab	2.37 ± 0.35b	5.79 ± 0.42c	1.00 ± 0.10a	2.13 ± 0.06b	1.75 ± 0.18b	2.66 ± 0.37b
Gluconeogenesis:								
* pck1*	1.00 ± 0.29	1.02 ± 0.24	1.34 ± 0.29	0.61 ± 0.30	1.00 ± 0.10	1.12 ± 0.05	0.95 ± 0.18	1.05 ± 0.39
* pck2*	1.00 ± 0.04a	2.10 ± 0.13b	2.09 ± 0.16b	1.99 ± 0.13b	1.00 ± 0.02a	2.34 ± 0.19b	1.90 ± 0.21b	2.49 ± 0.30b
* fbp1a*	1.00 ± 0.21a	1.80 ± 0.47ab	2.22 ± 0.22b	3.72 ± 0.40b	1.00 ± 0.05a	1.63 ± 0.19b	1.77 ± 0.04b	1.98 ± 0.18b
* fbp1b*	1.00 ± 0.07a	1.76 ± 0.19b	1.48 ± 0.28ab	1.06 ± 0.41ab	1.00 ± 0.03	1.11 ± 0.19	0.94 ± 0.08	1.19 ± 0.15
* g6pcb*	1.00 ± 0.25a	2.06 ± 0.38b	1.97 ± 0.21b	4.05 ± 0.54c	1.00 ± 0.13a	4.08 ± 0.70b	2.14 ± 0.19c	3.66 ± 0.39b
Glycogenolysis:								
* glycogen phosphorylase*	1.00 ± 0.19a	1.70 ± 0.13b	1.98 ± 0.20b	2.23 ± 0.21b	1.00 ± 0.12a	1.90 ± 0.23b	2.11 ± 0.28b	3.11 ± 0.36b
**Lipid metabolism**								
Fatty acid synthesis:								
* acly*	1.00 ± 0.09a	1.10 ± 0.18a	3.11 ± 0.29b	3.38 ± 0.25b	1.00 ± 0.08a	1.01 ± 0.17ab	1.42 ± 0.18b	3.13 ± 0.34c
* acaca*	1.00 ± 0.11a	1.07 ± 0.22a	2.91 ± 0.31b	2.06 ± 0.15b	1.00 ± 0.07a	0.97 ± 0.16a	1.60 ± 0.27b	1.86 ± 0.25b
* fasn*	1.00 ± 0.12a	1.84 ± 0.31b	1.48 ± 0.22b	1.73 ± 0.35b	1.00 ± 0.24a	2.18 ± 0.44b	3.00 ± 0.41b	2.96 ± 0.41b
Β-oxidation:								
* cpt1a*	1.00 ± 0.14a	1.12 ± 0.09a	1.81 ± 0.23b	1.99 ± 0.15b	1.00 ± 0.08a	0.79 ± 0.13a	1.84 ± 0.15b	1.81 ± 0.28b
* acadm*	1.00 ± 0.15a	0.61 ± 0.06b	0.66 ± 0.07b	0.62 ± 0.08b	1.00 ± 0.07a	0.62 ± 0.11b	1.01 ± 0.09a	0.94 ± 0.07a
* echs1*	1.00 ± 0.08a	0.86 ± 0.09ab	0.67 ± 0.04b	0.73 ± 0.07b	1.00 ± 0.09a	0.80 ± 0.07ab	0.77 ± 0.08b	0.62 ± 0.04b
* hadh*	1.00 ± 0.06a	0.99 ± 0.05a	0.77 ± 0.04b	0.78 ± 0.03b	1.00 ± 0.07a	0.83 ± 0.09ab	0.88 ± 0.09ab	0.65 ± 0.04b
Ketogenesis:								
* hmgcl*	1.00 ± 0.30a	1.79 ± 0.37a	5.46 ± 1.00b	6.73 ± 1.43b	1.00 ± 0.11a	2.63 ± 0.60ab	3.03 ± 0.47b	5.42 ± 0.94c
* acat1*	1.00 ± 0.09a	0.92 ± 0.08ab	1.55 ± 0.17b	2.77 ± 0.35c	1.00 ± 0.13a	1.30 ± 0.28a	2.74 ± 0.47b	4.03 ± 0.50c
**Transcriptional regulators**								
*ppargc1α*	1.00 ± 0.21a	1.72 ± 0.50ab	2.77 ± 0.35bc	3.71 ± 0.43c	1.00 ± 0.14a	2.30 ± 0.32b	2.35 ± 0.35b	2.41 ± 0.24b
*pparα*	1.00 ± 0.21a	1.51 ± 0.09b	2.38 ± 0.19c	2.42 ± 0.23c	1.00 ± 0.16a	1.99 ± 0.26b	1.69 ± 0.14b	2.23 ± 0.24b

Cells were incubated with culture media alone (control) or containing 0.1, 1 and 10 nM BDNF during 1 and 6 h. Data obtained by RT-qPCR are shown as mean + SEM of the results obtained in two different experiments (n = 6 in each experiment). Different letters indicate significant differences (p < 0.05) among groups within each time point assessed by one-way ANOVA and SNK test.

*acaca*, *acetyl-CoA carboxylase; acadm*, *acyl-CoA dehydrogenase medium-chain*; *acat1*, *acetyl-CoA acetyltransferase 1*; *acly*, *ATP citrate lyase*; BDNF, brain-derived neurotrophic factor; *cpt1a*, *carnitine palmitoyltransferase 1a*; *echs1*, *enoyl-CoA hydratase short-chain 1*; *fasn*, *fatty acid synthase*; *fbp1a*, *fructose 1,6-bisphosphatase 1a*; *fbp1b*, *fructose 1,6-bisphosphatase 1b*; *g6pcb*, *glucose 6-phosphatase b*; *gck*, *glucokinase*; *glut2*, *glucose transporter 2*; *hadh*, *3-hydroxyacyl CoA dehydrogenase*; *hmgcl*, *3-hydroxy-3-methylglutaryl-CoA lyase*; *pck1*, *phosphoenolpyruvate carboxykinase 1*; *pck2*, *phosphoenolpyruvate carboxykinase 2*; *pfkla*, *phosphofructokinase a*; *pfklb*, *phosphofructokinase b*; *pklr*, *pyruvate kinase*; *pparα*, *peroxisome proliferator-activated receptor alpha*; *ppargc1α*, *peroxisome proliferator-activated receptor gamma coactivator 1-alpha*; *sglt1*, *sodium-glucose cotransporter 1.*

### Levels of glucose transporters and the activity of key enzymes involved in glucose and lipid metabolism are modulated by BDNF in vitro

**Figure 5:**
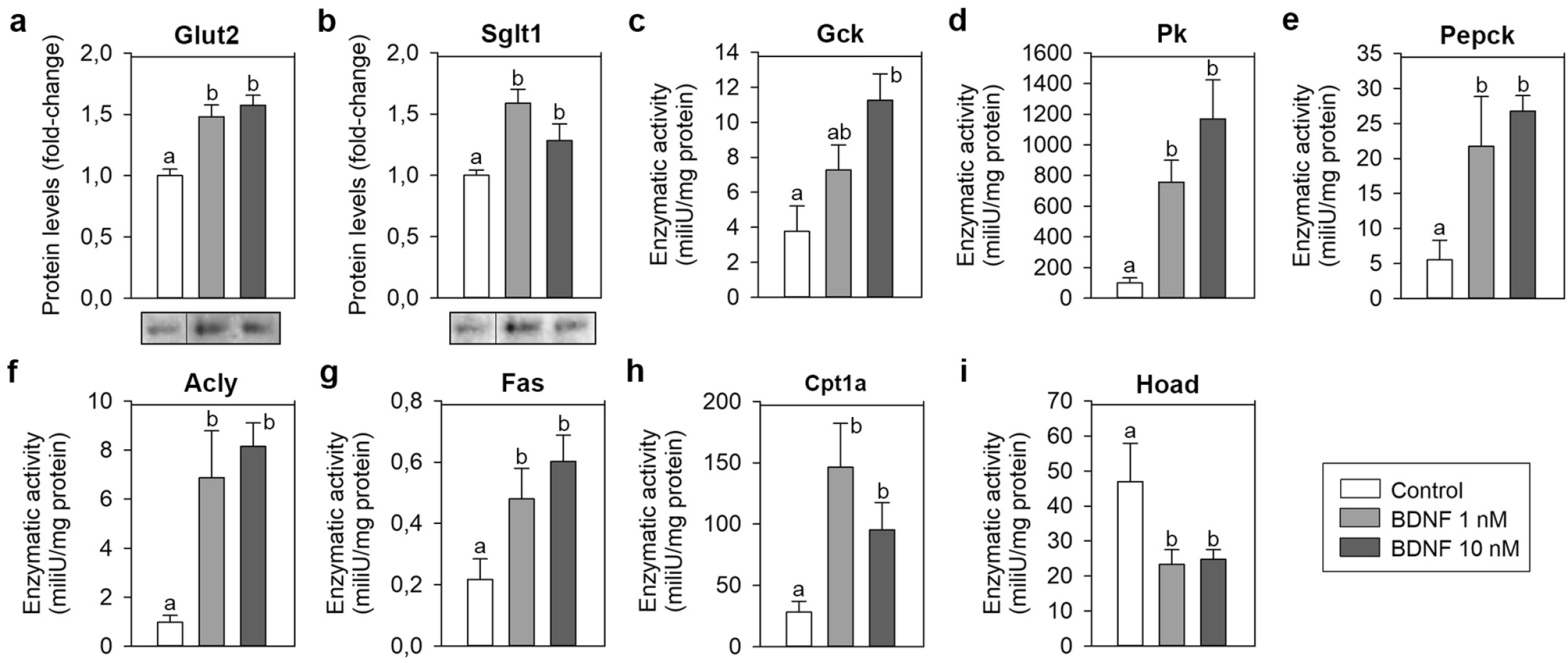
Effects of BDNF on the protein levels of Glut2 and Sglt1 (**a**,**b**) and on the activity of key enzymes involved in glucose and lipid metabolism (**c**–**i**) in ZFL cells. Cells were incubated with culture media alone (control) or containing 1 or 10 nM BDNF during 1 h. In figures (**a**,**b**), a representative blot is shown for each protein. Blots shown here were cropped from different parts of a same blot. Full-length blots are presented in SI Fig. S1. Data are shown as mean + SEM (n = 4 for **a**,**b**, and n = 8 for **c**–**i**). Different letters indicate significant differences (p < 0.05) among groups assessed by one-way ANOVA and SNK test. *Acly* ATP citrate lyase, *BDNF* brain-derived neurotrophic factor, *Cpt1a* carnitine palmitoyltransferase 1a, *Fas* fatty acid synthase, *Gck* glucokinase, *Glut2* glucose transporter 2, *Hoad* 3-hydroxyacyl CoA dehydrogenase, *Pepck* phosphoenolpyruvate carboxykinase, *Pk* pyruvate kinase, *Sglt1* sodium-glucose cotransporter 1.

Glut2 and Sglt1 proteins were upregulated (≈ 1.5-fold) in ZFL cells exposed to 1 and 10 nM BDNF during 1 h when compared to cells exposed to culture media alone [Glut2: ANOVA: F (2, 9) = 15.384, p = 0.001; SNK: p = 0.002 and 0.002; Sglt1: ANOVA: F (2, 9) = 7.711, p = 0.011; SNK: p = 0.009 and 0.048, respectively] (Fig. 5a,b). In vitro treatment with BDNF also resulted in a significant increase in the enzymatic activity of Gck [3.5-fold; ANOVA: F (2, 21) = 5.843, p = 0.01; SNK: p = 0.007], Pk [≈ 7.5- and 12-fold; ANOVA: F (2, 21) = 10.143, p < 0.001; SNK: p = 0.012 and < 0.001], Pepck [≈ 4- and 5-fold; ANOVA: F (2, 21) = 5.831, p = 0.01; SNK: p = 0.021 and 0.01], Acly [≈ 7- and 8-fold; ANOVA: F (2, 21) = 9.354, p = 0.001; SNK: p = 0.003 and 0.002], Fas (≈ 2- and 3-fold; ANOVA: F (2, 21) = 5.315, p = 0.014; SNK: p = 0.041 and 0.012] and Cpt1a [≈ 5- and 3.5-fold; ANOVA: F (2, 21) = 4.550, p = 0.023; SNK: p = 0.018 and 0.103] in ZFL cells (Fig. 5c–h). A significant reduction [≈ 2-fold; ANOVA: F (2, 21) = 3.661, p = 0.041; SNK: p = 0.045 and 0.034] in the activity of Hoad was observed in cells treated with BDNF (Fig. 5i).

## Discussion

BDNF has emerged as an important regulator of feeding and energy balance in mammals^40–42^. However, no evidence is available in fish regarding the existence of such a link between BDNF and the regulation of food intake and energy balance. The results of this research address this paucity of information in the fish literature. First, we studied whether BDNF and its receptors are present in tissues involved in the regulation of appetite and energy homeostasis (namely the brain, gut and liver) in zebrafish. The presence and distribution of the BDNF system in the zebrafish brain have been reported^2,12,14^. Thus, the presence of BDNF (both gene and protein) observed in this study in the zebrafish brain confirms these earlier findings. Our results also demonstrate that BDNF mRNA and protein are expressed in the liver and, although at low levels, are also present in the gut of zebrafish. This indicates the presence of BDNF in peripheral tissues. Interestingly, Western blot analysis detected not only a single band corresponding to the mature BDNF but also additional bands, which may correspond to different isoforms and/or glycosylated forms of BDNF and/or pro-BDNF. Indeed, three isoforms of pro-BDNF have been identified in zebrafish, with around 95% of identity^49^. The fact that different band patterns were observed among tissues might indicate a tissue-specific expression of such putative isoforms and/or post-translationally modified forms of BDNF in zebrafish. Besides BDNF, the presence of mRNAs encoding for receptors for this peptide (TrkB2, p75NTRa and p75NTRb) was also observed in the zebrafish gut and liver, supporting a putative role for the peptide in these tissues. Additionally, results from this study described that BDNF, TrkB2, p75NTRa and p75NTRb mRNAs and/or protein are present in other tissues of zebrafish, including the eye, gill, heart, spleen and muscle. This wide tissue distribution suggests a multifunctional role for the BDNF system in fish. It is worth mentioning that only the expression of *trkb2* (and not *trkb1 or other forms of this receptor*) was analyzed in this research. We chose to focus on *trkb2* based on previous studies suggesting that this isoform of TrkB is the main BDNF receptor in zebrafish, at least in the brain^27^ and the lateral line^28^. This is a limitation of our research, and future studies analyzing the expression pattern of other Trk receptors in zebrafish should be performed.

Second, we hypothesized that if BDNF regulates feeding, it might display periprandial variations in the expression and be regulated by food availability. Major findings from our study described an overall preprandial rise and a postprandial decrease in the expression of *bdnf* and its receptor mRNAs in the zebrafish foregut and liver. These periprandial profiles are typical of orexigenic peptides, including NPY^50^ and ghrelin^51,52^. However, we observed a postprandial increase in the abundance of *bdnf* and *trkb2* transcripts in the brain, which are in disagreement with observations in rest of the tissues. This may suggest a different biological response of the BDNF system to the peripheral nutritional status, or be linked to a different physiological action of this system in the zebrafish. The existence of periprandial variations in the expression of the BDNF system led us to the next study on whether BDNF administration influences appetite in zebrafish. Our results demonstrated that BDNF dose-dependently increases food intake in zebrafish at 1, 2 and 6 h post-IP injection. This result is concordant with the periprandial variations observed for the BDNF system in zebrafish peripheral tissues in this study. However, it differs from previous reports in mammals describing an anorexigenic role for BDNF^53–56^, which strongly suggests different physiological functions for BDNF between fish and mammals. Findings from the present study also indicate that the orexigenic role of BDNF in zebrafish may be mediated by the modulation of brain peptide circuitries, i.e. the upregulation of the orexigens NPY, AgRP and orexin, and the downregulation of the anorexigen NUCB2/nesfatin-1. Studies in mammals have shown that BDNF appears also to interact with NPY in its action on food intake, although in an opposite direction that the one described here given the anorexigenic action of BDNF in mammals^55^. In order to modulate central appetite-regulating hormones, peripherally administered BDNF must cross the blood–brain barrier. Although no studies are available in fish, BDNF was reported to be able to cross the blood–brain barrier in both directions in mammals^57^. BDNF actions in the zebrafish brain are likely mediated by the TrkB2 or p75NTRa receptors, but not p75NTRb, as suggested by the high expression of the two former ones and the lower expression of the latter in the hypothalamus. Besides brain circuitries, the increased expression of ghrelin in the foregut in response to 100 ng/g bw BDNF suggests that peripheral ghrelin might also contribute to the BDNF-induced increase in appetite in the zebrafish. While not conclusive, among the BDNF receptors studied here, only p75ntrb was detected in the zebrafish foregut, and so it could be a potential candidate that mediates the BDNF-induced upregulation of ghrelin expression. Notably, BDNF administration also caused a significant upregulation of brain *pomc* and *cart*, and of hepatic *leptin a* and *leptin b* mRNAs. Such observations may appear controversial given the anorexigenic nature of the peptides encoded by these three genes, and might be related to other functions of BDNF in zebrafish. In accordance with the role of BDNF in stimulating food intake in zebrafish, we also observed that expression of *bdnf* in the foregut is enhanced by food deprivation. Nevertheless, the effects of fasting were opposite in the brain and liver, where it downregulates the expression of the BDNF system. A similar effect of fasting on BDNF was described in the mammalian brain^58,59^. Based on our observations, it is plausible that the increased expression of *bdnf* in the foregut (directly implicated in sensing the levels of food intake) is enough for eliciting an increase in food intake in fasting states, while the reduced expression in the brain and gut might correlate with other effects of BDNF. However, further studies are needed to understand the physiological meaning of the fasting-evoked downregulation of the BDNF system in the zebrafish brain and liver.

Our next aim was to elucidate whether BDNF has a role in glucose and lipid metabolism in the zebrafish, as supported by the significant presence of the peptide and its receptor in the liver. Our in vivo and in vitro results demonstrated important changes in the expression and/or activity of metabolic enzymes in response to BDNF. The glucoregulatory role of BDNF in the zebrafish liver appears to be linked to increasing the levels of glucose in the hepatic cells, both by stimulating glucose entrance into the cells, or by gluconeogenesis and glycogen phosphorylation. The stimulation of glucose entrance into hepatocytes is suggested by the increase in mRNA and protein levels of the glucose transporters Glut2 and Sglt1 in vitro in response to BDNF exposure. The increased activity and/or mRNA expression of genes encoding the gluconeogenic enzymes Pepck, Fbpase and G6pase in vivo and in vitro points to an increase in gluconeogenesis. Finally, an increase in glycogen phosphorylation is suggested by the upregulation of *glycogen phosphorylase*. The role of BDNF in stimulating gluconeogenesis and glycogenolysis might be mediated by leptin, which is known to increase the expression of hepatic gluconeogenic enzymes^60^ and to induce glycogenolysis^61^ in fish. This would explain the increased mRNA expression of *leptin a* and *leptin b* in the zebrafish liver after IP administration of BDNF, which seems not to agree with the BDNF-evoked induction of food intake. The suggested capacity of BDNF to increase hepatic levels of glucose in the zebrafish seems to be contradictory with the reported effect of BDNF lowering blood glucose levels in mammals^45,46^. This points out once again that BDNF functions differently in fish and mammals, and warrant further studies on BDNF and its role on glucose homeostasis in vertebrates.

Interestingly, our results suggest that not only gluconeogenic but also glycolytic pathways are upregulated by BDNF. This is suggested by the increased expression of *pfkla* and *pfklb *in vivo, increased expression of *gck* and *pklr *in vitro, and increased activity of Gck and Pk in vitro. Together, such observations point to a situation in which gluconeogenesis and glycolysis appear to be simultaneously stimulated in the zebrafish hepatic cells in response to BDNF. This hypothesis needs, however, to be further confirmed, as our results in vivo showed no change in the levels of *gck*, and indeed decreased levels of *pklr* mRNA, in response to BDNF administration. It is possible that BDNF is simultaneously stimulating gluconeogenesis and glycolysis directly in the liver, but in an in vivo situation where multiple factors are interacting in the modulation of physiological processes, the result is an inhibition of the glycolytic pathways. Gluconeogenesis and glycolysis are typically regulated in order not to occur at the same time, however the simultaneous stimulation of both processes have been previously reported in a fish species^62^. If both gluconeogenesis and glycolysis are being upregulated by BDNF, it could be hypothesized that the high amount of glucose that is entering into the hepatic cells and that is being synthesized in response to BDNF is at the same time being hydrolyzed. Glucose hydrolysis could be related to either the obtention of energy through the Krebs cycle, or to the synthesis of fatty acids.

An increase in fatty acid synthesis in response to BDNF is suggested by our results, given the increased expression and/or activity of the enzymes Acly, Acc and Fas in BDNF-treated groups compared to control groups. Therefore, it would be possible that the pyruvate that results from the glycolysis and is converted into citrate, instead of being used for the Krebs cycle, is being released into the cytoplasm and used as a substrate for fatty acid biosynthesis^63^. Thus, in the cytoplasm, citrate would be converted into acetyl-CoA by Acly, then into malonyl-CoA by Acc and finally into palmitate by Fas. Palmitate can be either β-oxidized in order to obtain more energy or be used for synthesizing phospholipids for maintaining cell membrane structure. Previous reports in mammals indicated that only 20–30% of palmitate is β-oxidized, while about 60–70% of palmitate in the liver and most bodily tissues is incorporated into phospholipids^64^. Results from the present study seem to be in accordance with such an effect in mammals, as the reduced expression of *acadm*, *echs1* and *hadh* by BDNF suggests that β-oxidation is being inhibited by this peptide in the zebrafish liver. Liver-specific BDNF mutant mice contain the same expression levels of *Acadl* (*long-chain acyl-coenzyme A dehydrogenase*) and *Acadm* compared to wild-type mice when fed a standard chow, but higher levels were found when fed a high-fat diet^65^. This suggests that BDNF does not regulate β-oxidation under a normal diet in mammals, but might diminish the effects of enhanced lipid oxidation, ultimately resulting in hepatic steatosis, under a high-fat diet challenge. While our gene expression results point to a situation in which BDNF would inhibit β-oxidation in zebrafish fed a normal diet, it would be interesting to study the effects of BDNF in fish fed a high-fat diet. In our study, the increase in the mRNA levels and activity of Cpt1a, in charge of facilitating the entrance of fatty acids into the mitochondria, appears to be in disagreement with BDNF not promoting β-oxidation. To study whether this putative increase in the entrance of fatty acids into the mitochondria might be instead associated with an increase in the formation of ketonic bodies, we studied the expression of key enzymes involved in such process. Levels of *hmgcl* and *acat1* mRNAs were upregulated by BDNF in vivo and in vitro, supporting a BDNF-derived induction of ketogenesis in the zebrafish liver. No previous reports are available on the direct effects of BDNF on ketogenesis. However, it has been described that a ketogenic diet alters BDNF expression, although both an upregulation^66^ and a downregulation^67^ were reported. This observation in mammals together with our results might suggest a reciprocal regulatory loop between BDNF and ketogenesis, although further studies are required, especially given the differences we are here describing between the physiology of BDNF in fish and mammals. Ketogenesis is a typical response to a situation of very low glucose levels and depletion of glycogen stores, such as a prolonged fasting^68^. While we do not have evidence for any cellular metabolic changes after BDNF, the indications from the gene expression changes affecting metabolic machinery suggest an increase in hepatic glucose levels and ketogenesis in response to BDNF. This might suggest that BDNF mimics a metabolic state similar to the one induced by fasting in the zebrafish. This is in accordance with observations in mammals reporting that BDNF mediates the adaptive responses of brain and body to energetic challenges (e.g., food deprivation and exercise)^40^. Based on the tissue distribution profiles shown in this study, all proposed actions for BDNF in the liver are likely mediated by TrkB2 and/or p75ntra, but not p75ntrb. Nevertheless, further studies are needed to confirm the involvement of the different receptor subtypes in the actions of BDNF in the zebrafish.

Finally, we investigated whether Ppargc1*α* and Pparα mediate the actions of BDNF in the zebrafish liver. PPARGC1*α* is a key transcriptional regulator of energy homeostasis known to stimulate the transcription of genes involved in gluconeogenesis, Krebs cycle flux, fatty acid oxidation and mitochondrial oxidative phosphorylation in mammals^69–71^. PPARα acts as a ligand-activated receptor, controlling the transcription of genes involved in lipid homeostasis^72,73^. In mammals, it has been shown that BDNF is a downstream of PPARGC1*α*^74–76^, but whether BDNF also activates such coactivator remains to be studied. Present observations showed that *ppargc1α* and *pparα* mRNA levels are upregulated by in vivo and in vitro treatment with BDNF in the zebrafish liver, suggesting that BDNF actions in this tissue are mediated (at least partially) by the induction of these two transcriptional regulators.

In summary, this study described the presence of the BDNF system in several tissues of zebrafish, some of which are key regulators of appetite and energy homeostasis. Indeed, we demonstrated that BDNF is an important meal-responsive orexigen in zebrafish. This is the first study reporting the role of BDNF in food intake in fish, and its periprandial profiles in a vertebrate. Additionally, we provided evidence in favor of an important involvement of BDNF in glucose and lipid metabolism in the zebrafish liver, by acting on the expression and/or activity of enzymes implicated in key processes regulating energy balance. Such roles of BDNF in energy homeostasis points to an increase in hepatic glucose levels, lipogenesis and ketogenesis. However, further studies analyzing changes in metabolite levels in response to BDNF are needed to corroborate this putative catabolic role for BDNF in the zebrafish liver. This is especially true considering the fact that BDNF has an orexigenic role in zebrafish. Results presented here describe novel aspects on the physiology of BDNF of both fish and mammals, and add significant new information to our growing knowledge on regulators of metabolic and endocrine functions in vertebrates. Future lines of investigation should evaluate the metabolic effects of BDNF under a high-fat diet challenge, and should focus on the mechanism of action and signaling pathways activated by BDNF.

## Methods

### Animals

Zebrafish (*Danio rerio*), with a body weight (bw) of ~ 1 g, were obtained from the Aquatic Toxicology Research Facility at the University of Saskatchewan and housed in 10 L aquaria with a constant flow of temperature-controlled water (26 ± 1 °C). Fish were maintained under a 12 h light:12 h darkness (12L:12D) photoperiod (lights on at 07:00 h), and fed daily at 11:00 h with commercial slow-sinking pellets (1% bw; Aqueon Catalog# 06053, Franklin, WI, USA). For all experiments using zebrafish, fish were anesthetized using tricaine methanesulfonate (MS-222; Syndel Laboratories, Nanaimo, BC, Canada) and euthanized by decapitation. All fish studies complied within the policies of the Canadian Council for Animal Care, and were approved by the University of Saskatchewan Animal Research Ethics Board (Protocol Number 2012–0082).

### ZFL cells

Zebrafish liver (ZFL) cells were purchased from ATCC (Catalog # ATCC^®^ CRL-2643™; Manassas, VA, USA) and cultured at 28 °C under a 100% air atmosphere. The media used for cell culture had the following components: 50% Leibovitz’s L-15 (Thermo Fisher Scientific, Waltham, MA, USA), 35% Dulbecco’s Modified Eagle Medium (DMEM) High Glucose (Sigma-Aldrich, Oakville, ON, Canada) and 15% Ham's F12 (ATCC) (all without sodium bicarbonate), supplemented with 0.15 g/L sodium bicarbonate, 15 mM HEPES, 0.01 mg/mL bovine insulin, 50 ng/mL mouse epidermal growth factor (EGF), 5% heat-inactivated fetal bovine serum and 0.5% trout serum. For studies, ZFL cells were seeded at 5 × 10^5^ cells/well in 24-well plates or 1 × 10^6^ cells/well in 6-well plates, and grown at 80–90% confluency (typically 48–72 h after seeding) before performing the experiment.

### Experimental design

#### Tissue distribution of BDNF and its receptors

The distribution of the BDNF system within the zebrafish was studied both at mRNA and protein levels. For mRNA, the following tissues were collected from six zebrafish: brain (without the hypothalamus), hypothalamus, eye, skin, gills, heart, foregut (intestinal bulb and anterior most portion of the intestine, ≈ 0.5 cm), hindgut (posteriormost portion of the intestine, ≈ 0.5 cm), liver, spleen, ovary, testis and muscle. Tissues were immediately frozen in liquid nitrogen and stored at − 80 °C until quantification of gene expression (see “Quantification of mRNA abundance by real-time quantitative PCR (RT-qPCR)”). For protein determination, samples of brain, hypothalamus, eye, gill, foregut, liver and spleen were dissected, frozen in liquid nitrogen and stored at − 80 °C until Western blot analysis (see “Determination of protein levels by Western blot”). Additionally, two samples of foregut and liver were collected and immediately transferred to 4% paraformaldehyde for immunohistochemistry aimed to determine BDNF-like immunoreactivity (see “Detection of BDNF-like immunoreactivity by immunohistochemistry”).

#### Periprandial expression of the BDNF system

Fish were divided into seven groups (n = 6/group) and acclimated for 2 weeks. On the day of the experiment, samples of whole brain, liver and foregut were collected at 3 h prior to feeding (− 3 h), 1 h prior to feeding (− 1 h), at the regular feeding time (0 h), 1 h after feeding (+ 1 h) and 3 h after feeding (+ 3 h). Two groups remained unfed and were sampled at + 1 h and + 3 h. Tissue samples were collected and stored at − 80 °C for mRNA expression analysis (see “Quantification of mRNA abundance by real-time quantitative PCR (RT-qPCR)”).

#### Fasting-induced changes in the mRNA expression of the BDNF system

Two groups of fish (n = 6/group) were acclimated to tank conditions during 2 weeks. One group of fish was then not fed or exposed to food for 7 days, while the other group continued to receive food at the regular feeding time. At the end of 7 days, brain, liver and foregut were sampled at 11:30 h from both fed and fasted fish. The expression of BDNF system mRNAs was quantified as described below (see “Quantification of mRNA abundance by real-time quantitative PCR (RT-qPCR)”).

#### Effects of BDNF on food intake and on the expression of appetite regulators and genes involved in glucose and lipid metabolism

Twelve groups of fish (n = 3/group) were acclimated to tank conditions for 3 weeks. For one week prior to the experiment, daily basal food intake in each tank was recorded. For this, a pre-weighed amount of food was offered to fish in each tank. After 30 min, the uneaten food was collected, dried for 24 h and weighed. Quantification of food intake was determined by subtracting the dry weight of the amount of food retrieved from the tank after 30 min of feeding from the dry weight of the total amount of food originally provided. For each group of fish, levels of food intake during the 3 days prior to the experiment were averaged and considered the baseline food intake. On the day of experiment, fish were anesthetized prior to their daily scheduled feeding time and intraperitoneally (IP) injected with either sterile saline (0.9% NaCl; control group) or saline containing 1, 10 or 100 ng/g bw of BDNF (Recombinant human BDNF, Catalog # ab206642; Abcam, Toronto, ON, Canada). Of the total of 12 fish groups initially set up, 3 groups were used as control and 3 groups were used for each of the BDNF dose. Even though peptide used here is human BDNF, it was selected based on a BLAST analysis that showed 91.6% identity with the zebrafish peptide. After injections, fish were allowed to recover (5 min) and were fed a pre-weighed quantity of food. Uneaten food pellets were removed 1 h post-feeding, and the amount of food intake was calculated as described above. At 2 h post-injection, a new amount of pre-weighed food was offered again to fish, and, after 30 min, uneaten pellets were collected to calculate food intake levels as described above. The same procedure was repeated at 6 and 24 h post-injection. Food intake levels were calculated for every group of fish (tank) as percentage of food ingested with respect to their corresponding baseline levels (considered as 100%). The complete experiment was repeated three times. At the fourth repetition, injected fish were only allowed to eat at 2 h post-injection. Then, fish were anaesthetized again, sacrificed by decapitation, and brain, foregut and liver were collected. Samples were kept at − 80 °C until gene expression analysis (see “Quantification of mRNA abundance by real-time quantitative PCR (RT-qPCR)”).

#### Effects of BDNF on the mRNA levels of genes involved in glucose and lipid metabolism in vitro

ZFL cells were seeded at 5 × 10^5^ cells/well in 24-well plates and grown to confluency as described earlier. Once 80–90% confluency was achieved, media was replaced by 1 mL of fresh media alone (6 wells) or containing BDNF (0.1, 1 or 10 nM; 6 wells each). After an incubation period of 1 h and 6 h, media was removed and 500 µL of PureZOL™ RNA Isolation Reagent (Bio-Rad, Mississauga, ON, Canada) was added to each well. Cells were then scraped, transferred to tubes and stored at − 80 °C until total RNA was extracted (see “Quantification of mRNA abundance by real-time quantitative PCR (RT-qPCR)”). This experiment was repeated twice.

#### BDNF effects on the abundance of glucose transporters and the activity of enzymes implicated in glucose and lipid metabolism in vitro

For this assay we chose the concentrations and time in which BDNF exerts the most significant inductions in mRNA expression. ZFL cells were seeded at 1 × 10^6^ cells/well in 6-well plates and grown to confluency. Then, culture media was replaced by 1 mL of fresh media alone (4 wells for Western blot and 8 wells for assessment of enzymatic activity) or containing 1 or 10 nM BDNF (4 wells each for Western blot and 8 wells each for assessment of enzymatic activity), and plates were incubated for 1 h. At the end of the culture time, media was removed and 300 µL of lysis buffer was added to each well. For Western blot analysis, the lysis buffer used was T-PER Tissue Protein Extraction Reagent (Thermo Fisher Scientific), while samples for enzymatic activity assessment were lysed in a 80 mM Trizma buffer (pH 7.6) containing 5 mM EDTA, 2.6 mM DTT and protease inhibitor cocktail (Thermo Fisher Scientific). After the addition of the buffer, cells were scraped, collected and frozen at − 80 °C until further analysis (see “Determination of protein levels by Western blot”, “Enzymatic activity determination”).

### Quantification of mRNA abundance by real-time quantitative PCR (RT-qPCR)

Total RNA was isolated using PureZOL™ RNA Isolation Reagent (Bio-Rad). RNA purity was validated by optical density (OD) absorption ratio (OD 260 nm/280 nm) using a NanoDrop 2000c (Thermo, Vantaa, Finland). Then, an aliquot of 1 µg of total RNA was reverse transcribed into cDNA in a 20 µL reaction volume using iScript Reverse Transcription Supermix for RT-qPCR (Bio-Rad, Mississauga, ON, Canada) according to the manufacturer’s instructions. Real-time quantitative PCRs were performed using SensiFAST SYBR No-ROX Kit (FroggaBio, Toronto, ON, Canada). The specific primer sequences used for target genes, and reference gene (*β-actin*) are shown in SI Table S1 and were ordered from IDT (Toronto, ON, Canada). Genes were amplified in duplicated RT-qPCR runs using a 96-well plate loaded with 1 µL of cDNA and 500 nM of each forward and reverse primer in a final volume of 10 µL. Each PCR run included a standard curve for the corresponding gene made of two replicates of four serial dilution points, and water instead of cDNA as control in order to ensure that the reagents were not contaminated. RT-qPCR cycling conditions consisted of an initial step of 95 °C for 3 min, and 35 cycles of 95 °C for 10 s and 60 °C for 25 s. A melting curve was systematically monitored (temperature gradient at 0.5 °C/5 s from 65 to 95 °C) at the end of each run to confirm specificity of the amplification reaction. All runs were performed using a CFX Connect Real-Time System (Bio-Rad). The 2-ΔΔCt method^77^ was used to determine the relative mRNA expression.

### Determination of protein levels by Western blot

Proteins were extracted from tissues and cells using T-PER Tissue Protein Extraction Reagent (Thermo Fisher Scientific) as directed by the manufacturer. Bradford assay (Bio-Rad) was used to determine protein concentration. The samples (containing 20 µg protein) were prepared in 4× Laemmli buffer containing 0.2% of 2-mercaptoethanol (Bio-Rad) and were subjected to boiling at 95 °C for 10 min prior to loading. Samples were then run on a gradient gel (Bio-Rad) and transferred to a 0.2 μm nitrocellulose membrane (Bio-Rad). After blocking using 1× RapidBlock solution (AMRESCO, Toronto, ON, Canada), target proteins within the membrane were detected by overnight incubation with specific primary antibody: mouse monoclonal to BDNF (1:500 dilution; Catalog # ab203573; Abcam), goat polyclonal to GLUT2 (1:500 dilution; Catalog # ab111117; Abcam) and rabbit polyclonal to SGLT1 (1:500 dilution; Catalog # ab14686; Abcam). Vinculin protein was used for normalization and was detected using rabbit antiserum directed against mouse vinculin (1:1,000 dilution; Catalog # ab129002, Abcam). Secondary antibodies used were: sheep anti-mouse, goat anti-rabbit or rabbit anti-goat IgG (H+L) HRP conjugate (1:2,000 dilution; Bio-Rad). For visualization of protein, the membrane was incubated for 5 min in Clarity Western ECL substrate (Bio-Rad) and imaged using ChemiDoc MP imaging system (Bio-Rad). Blot images were analysed using ImageLab software and band density of vinculin was used to normalize glucose transporter protein density.

### Detection of BDNF-like immunoreactivity by immunohistochemistry

Samples from zebrafish foregut and liver were collected as previously described and processed (dehydrated and embedded in paraffin) at the Prairie Diagnostic Services, University of Saskatchewan. Paraffin blocks were then sectioned at 7 µm thickness, and transverse sections were mounted onto Superfrost slides (Thermo Fisher Scientific). The protocol for IHC was performed as previously described^78^. Mouse monoclonal human BDNF antibody (1:200 dilution; Catalog # ab203573; Abcam) was used to detect BDNF-like immunoreactivity. As with the peptide, even though this antibody is specifically designed to react with mammalian species, a BLAST analysis of the immunogen showed more than 90% identity with BDNF zebrafish sequence. Additionally, Western blot analysis here performed detected a band of the expected size. Nevertheless, since it is likely that a certain degree of non-specificity exists in our findings, the suffix “-like” was used to refer to immunostaining obtained. Secondary antibody used was goat anti-mouse IgG Alexa Fluor 488 (Invitrogen, Burlington, ON, Canada). Separate sets of slides were used for negative and preabsorption controls. Negative control slides were only treated with the secondary antibody. Preabsorption controls were carried out by incubating slides with a preabsorption mixture of BDNF and primary antibody, using a protocol previously described^79^. All primary and secondary antibodies were diluted in an antibody diluent (Dako, Mississauga, ON, Canada). Slides were mounted using VECTASHIELD mounting medium containing 4′,6-diamidino-2-phenylindole (DAPI; Vector Laboratories, Burlington, ON, Canada) and assessed using a Nikon Eclipse Ti-Inverted fluorescence microscope (Nikon Instruments, Melville, NY, USA) connected to a Nikon DS-Qi1 MC camera. Micrographs were adjusted linearly for light and contrast using Photoshop CS6 (Adobe Systems Inc., San Jose, CA, USA).

### Enzymatic activity determination

The activity of glucokinase (Gck; Enzyme Commission, EC, number 2.7.1.2), pyruvate kinase (Pk; EC 2.7.1.40), phosphoenolpyruvate carboxykinase (Pepck; EC 4.1.1.32), ATP citrate lyase (Acly; EC 4.1.3.8), fatty acid synthase (Fas; EC 2.3.1.85), carnitine palmitoyltransferase 1a (Cpt1a; EC 2.3.1.21) and 3-hydroxyacyl CoA dehydrogenase (Hoad; EC 1.1.1.35) was assessed following protocols previously described for rainbow trout by Soengas et al.^80,81^, with slight variations for their use in ZFL cells. Briefly, enzymatic activities were determined in 96-well plates loaded with ZFL homogenates (10–50 μL) and 160–270 µL reaction buffer (omitting the substrate in control wells). Composition of the reaction buffers used for the enzymes tested is as follows. Gck buffer consisted in a Trizma buffer (80 mM, pH 8.0) containing 10.2 mM KCl, 37.5 mM MgCl_2_, 11.5 mM KH_2_PO_4_, 20 mM NaHCO_3_, 4 mM EDTA, 2.6 mM DTT, 2 mM NADP^+^, 7 mM ATP, 0.13 U mL^−1^ glucose 6-phosphate dehydrogenase, 0.13 U mL^−1^ 6-phosphogluconate dehydrogenase, and 1.2 M and 20 mM d-glucose (omitted for controls). Pk buffer was an imidazole buffer (50 mM, pH 7.4) containing 100 mM KCl, 10 mM MgCl_2_, 0.5 mM ADP, 0.15 mM NADH, 22 U mL^−1^ lactate dehydrogenase, and 1.5 mM phosphoenolpyruvate (omitted for controls). Pepck was assessed in a Trizma buffer (50 mM, pH 7.5) containing 1 mM MnCl_2_, 20 mM NaHCO_3_, 1.5 mM phosphoenolpyruvate, 0.3 mM NADH, 1.8 U mL^−1^ malate dehydrogenase, and 0.5 mM 2′-deoxyguanosine-5-diphosphate (omitted for controls). Acly was assessed in a Trizma buffer (50 mM, pH 7.8) containing 100 mM KCl, 10 mM MgCl_2_, 20 mM citric acid, 10 mM β-mercaptoethanol, 5 mM ATP, 0.3 mM NADH, 7.4 U mL^−1^ malate dehydrogenase, and 500 μM Coenzyme A (omitted for controls). Fas buffer consisted in a phosphate buffer (0.1 mM K_2_HPO_4_ and 0.1 mM KH_2_PO_4_, pH 6.5) containing 0.1 mM NADPH, 25 μM acetyl-CoA, and 100 μM malonyl-CoA (omitted for controls). Cpt1a was determined in a Trizma buffer (75 mM, pH 8.0) containing 1.5 mM EDTA, 0.25 mM 5,5′-dithiobis(2-nitrobenzoic acid) (DTNB), 50 μM palmitoyl-CoA, and 2 mM l-carnitine (omitted for controls). Finally, Hoad was assessed in an imidazole buffer (50 mM, pH 7.6) containing 0.15 mM NADH, and 300 μM acetoacetyl-CoA (omitted for controls). Once plates were loaded, reactions were allowed to proceed at 37 °C for pre-established times (3–25 min). Reaction rates of enzymes were determined by the decrease in absorbance of NADH at 340 nm (in the case of Pk, Pepck, Acly, Fas and Hoad), the increase in absorbance of NADPH at 340 nm (Gck), or the increase of DTNB-CoA complex at 412 nm (Cpt1a). All measurements were carried out in a SpectraMax 190 microplate reader (Molecular Devices, San Jose, CA, USA). Enzyme activities are expressed per mg protein, which was assayed by Bradford assay (Bio-Rad).

### Statistical analysis

Statistical differences between groups were assessed using either Student’s t test (for comparisons between two groups) or one-way ANOVA followed by Student–Newman–Keuls (SNK) multiple comparison test (for comparisons among multiple groups), after data were checked for normality and homogeneity of variance. Data that failed one of these requirements were log-transformed and re-checked. Significance was assigned when p < 0.05. All analyses were carried out using SigmaPlot version 12.0 (Systat Software Inc., San Jose, CA, USA) statistics package.

## Supplementary information

Supplementary information
