# Supplementary material for: Feeding and food availability modulate brain-derived neurotrophic factor, an orexigen with metabolic roles in zebrafish
Source: Sci Rep. 2020 Jul 1;10:10727. doi: 10.1038/s41598-020-67535-z (PMC7329848; doi:10.1038/s41598-020-67535-z)
Supplement: Supplementary file 1 — Supplementary information [file 41598_2020_67535_MOESM1_ESM.pdf]

# Feeding and Food Availability Modulate Brain-Derived Neurotrophic Factor, an Orexigen with Metabolic Roles in Zebrafish

Ayelén Melisa Blanco[1,2], Juan Ignacio Bertucci[1,3], Azadeh Hatef[1,3,] Suraj Unniappan[1*]

[1] Laboratory of Integrative Neuroendocrinology, Department of Veterinary Biomedical Sciences, Western College of Veterinary Medicine, University of Saskatchewan, Saskatoon, Saskatchewan, Canada.

[2] Laboratorio de Fisioloxía Animal, Departamento de Bioloxía Funcional e Ciencias da Saúde, Facultade de Bioloxía and Centro de Investigación Mariña, Universidade de Vigo, Vigo, Pontevedra, Spain.

[3] Toxicology Centre, University of Saskatchewan, Saskatoon, Saskatchewan, Canada.

**Supplementary Figure 1.** Full-length images of immunoblots included in Figures 5a and b. Bands shown in Figures 5a and b are boxed. For each protein, two separate blots containing two samples per treatment were performed. Figure here shows one of those two blots. **(a)** Glut2. **(b)** Sglt1. Molecular markers are included. See corresponding figure legend for details.

**a**

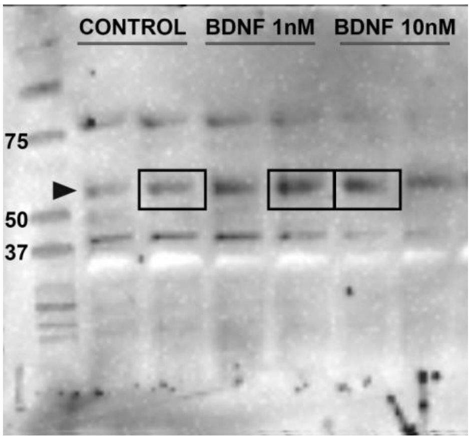


**b**

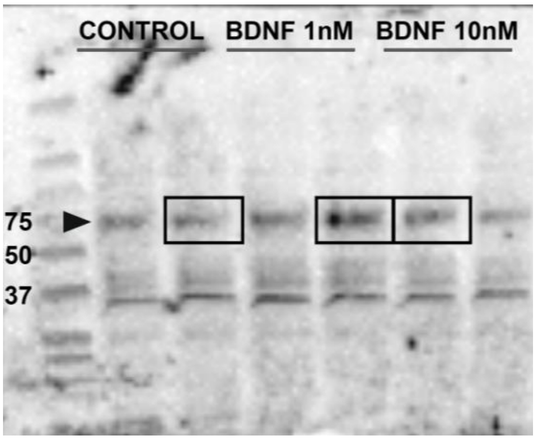

**Supplementary Table S1.** Primers used for quantifying gene expression by RT-qPCR in this study.

| Gene | GenBank accession number | Primer sequence (5' to 3') | Product size (bp) |
|---|---|---|---|
| *β-actin* | AF057040.1 | F: TTCAAACGAACGACCAACCT<br>R: TTCCGCATCCTGAGTCAATG | 93 |
| *acaca* | NM_001271308.1 | F: AATCAGGTGGTACGGATGGC<br>R: GGATGTTCCCTCTGTTGGGG | 160 |
| *acadm* | NM_213010.2 | F: AAGGTTTTGAGGGCAGGTGT<br>R: ACTCTTTCTGCTGCTCGGTT | 139 |
| *acat1* | NM_001003746.1 | F: ATCCCGCAGAGAGGAAAACC<br>R: CGAGTGGTGTGACGTTGAGT | 211 |
| *acly* | BC076484.1 | F: CCCACACCGCTAACTTCCTT<br>R: TCCTGGCGCGAACAAACATA | 247 |
| *agrp* | NM_001328012.1 | F: CTGGGACGTGAGCACTACAG<br>R: AAGGTGCTCCATTTCAGGCA | 169 |
| *bdnf* | U42489.1 | F: CGAGGAATAGACAAGCGGCA<br>R: ATCCGTATAAACCGCCAGCC | 110 |
| *cart* | GU057836.1 | F: GAGAGACTTGGCTGAGGCAC<br>R: GAAAGTGTTGCAGGCGGTTC | 181 |
| *cck* | XM_001346104.6 | F: ACGCTGGACTCTGTGTAT<br>R: CTTCATCGTCCTCTGGTTTG | 186 |
| *cpt1a* | NM_001044854.1 | F: TCTACCTGAGAGGTCGTGGG<br>R: TAGCCGTTCCCATTGAGCAG | 215 |
| *echs1* | NM_001004529.2 | F: CCCTTGCGATGGAGATGGTT<br>R: CGACTGCCCTCAGCTAAAGT | 214 |
| *fasn* | XM_009306807.3 | F: GGCGAGTGGTCAGTCAGTTA<br>R: TTGTTCGACTCAGGAAGGCG | 134 |
| *fbp1a* | NM_199942.2 | F: TGGCGAGTTCATTCTGGTGG<br>R: TCTGCCACCATTGAGCCTAC | 183 |

| | | | |
|---|---|---|---|
| *fbp1b* | NM_213132.1 | F: GAGTCCCAAGGGCAAGCTAA<br>R: TACAGGAACCCTCTGGTGGA | 142 |
| *g6pcb* | XM_002661194.6 | F: CATCTGGACACCACACCCTT<br>R: TGGGTGGTCTGAACGAGTCT | 196 |
| *gck* | NM_001045385.2 | F: GACACAGGGGACAGAAAGCA<br>R: CCACCCCCACAGTGATCTTT | 205 |
| *glut2* | DQ098687.1 | F: GGATACAGCTTGGGCGTCAT<br>R: CTCTGTGCCATTTCCCCCTT | 117 |
| *glycogen phosphorylase* | AY576991.1 | F: TGTAAAGTCCTCGCGCACA<br>R: ACATCCCCGAGTCCTGCTAT | 156 |
| *grl* | AM055940.1 | F: ATGGTCCCGTGCTTCAGAAT<br>R: TGCACCCACTTTGCTACAGA | 206 |
| *hmgcl* | NM_201215.1 | F: AGCCACGTCAATAGAAAGCAGT<br>R: CCTGGTCCTTTTGCCTCTGT | 157 |
| *hadh* | NM_001003515.1 | F: TGGAGGCTGTTCGGTTACAC<br>R: TTTGTTGAGCAGGGGACTGG | 203 |
| *leptin a* | NM_001128576.1 | F: GCTCTCCGCTCAACCTGTATT<br>R: TTTGCCCGTCAATGTGTTCC | 136 |
| *leptin b* | NM_001030186.3 | F: TTCCCCGTCACCTCCAACTA<br>R: CCTTGCATGTGCCATTGTGTT | 190 |
| *npy* | NM_131074.2 | F: GGCCACCAGATCTCATAAA<br>R: GCGCACATTGACGTATTT | 146 |
| *nucb2 a* | NM_201493.1 | F: AGGAGCGGCATGAAGAATTT<br>R: GATGGTTGACTTTGGGGTGA | 156 |
| *nucb2 b* | NM_201479.1 | F: TCTGTGGGCTTGTTTGGATG<br>R: TTCTCTCTGAAATGCGGGTC | 168 |
| *orexin* | NM_001077392.2 | F: AGAAACGACTCTTCCGTCGC<br>R: CGGCTTGATTCCGTGAGTTG | 148 |
| *p75ntr a* | XM_003198085.5 | F: GAGTTGGCAAGCTCTTGTGC<br>R: AAGTGAAGCTCAGCCATCGG | 233 |

| | | | |
|---|---|---|---|
| *p75ntr b* | NM_001198660.1 | F: TACAACCAGGGGAGAGTGCT<br>R: CATCATTGGCATCCGTGCAG | 203 |
| *pck1* | NM_214751.1 | F: AGCTCTTCAGGGTCTCGCA<br>R: TAACGTGTGTGTTGCGTGTCTT | 185 |
| *pck2* | NM_213192.1 | F: TCCTTCGGCAGTGGTTATGG<br>R: GCTGCTGCAATGTACCGTTT | 161 |
| *pfkla* | XM_693543.8 | F: AGGTATGAACGCAGCCATCC<br>R: TGCCAATCACTGTTCCTCCC | 173 |
| *pfklb* | NM_001328389.1 | F: TTTGAGCACAGGATGCCGAA<br>R: TCGATGCTAAGGGTTCGACG | 140 |
| *pklr* | BC152219.1 | F: CCAGTTTAACACGCGCGGC<br>R: GGGAAGTGTCCTTTGGCTGT | 113 |
| *pomc* | NM_181438.3 | F: CACTGCTCACACTCTTCA<br>R: GCCCACCTTCGTTTCTAT | 167 |
| *pparα a* | NM_001161333.1 | F: TAATCCACTCTCTGCGGCTC<br>R: CATGTTACTGCCGGTCTCCT | 247 |
| *ppargc1α* | ENSDART00000097710.6 | F: AAAGCCGGTGAAGCCAAGAG<br>R: GGTCACTGCAACACAGAGGA | 238 |
| *sglt1* | NM_200681.1 | F: TGTCCGTCATGTTGGCTTCA<br>R: TCTGAGCCGTCTGAACGATG | 198 |
| *trkb2* | NM_001197161.2 | F: TCACCTATGGCAAGCAACCC<br>R: CTTTGGGGCAAGTACGAGGT | 102 |

F, Forward primer; R, Reverse primer

*acadm, medium-chain acyl-CoA dehydrogenase; acat1, acetyl-CoA acetyltransferase 1; acc, acetyl-CoA carboxylase; acly, ATP citrate lyase; agrp, agouti-related protein; bdnf, brain-derived neurotrophic factor; cart, cocaine- and amphetamine-regulated transcript; cck, cholecystokinin; cpt1a, carnitine palmitoyltransferase 1a; ech, enoyl-CoA hydratase; fas, fatty acid synthase; fbpase 1a, fructose bisphosphatase 1a; g6pase b, glucose 6-phosphatase b; gk, glucokinase; glut2, glucose transporter 2; grl, ghrelin; hmgcl, 3-hydroxy-3-methylglutaryl-CoA lyase; hoad, 3-hydroxyacyl CoA dehydrogenase; npy, neuropeptide Y; nucb2, nucleobindin 2; p75ntr, neurotrophin receptor p75; pepck2, phosphoenolpyruvate carboxykinase 2; pk, pyruvate kinase; pomc, proopiomelanocortin; pparα, peroxisome proliferator-activated receptor alpha;*

*ppargc1α, peroxisome proliferator-activated receptor gamma coactivator 1-alpha; sglt1, sodium-glucose cotransporter 1; trkb2, tropomyosin receptor kinase B2.*
